# Phosphonodiamidate prodrugs of phosphoantigens (ProPAgens) exhibit potent Vγ9/Vδ2 T cell activation and eradication of cancer cells†

**DOI:** 10.1039/d4md00208c

**Published:** 2024-06-03

**Authors:** Qin Xu, Maria Sharif, Edward James, Jack O. Dismorr, James H. R. Tucker, Benjamin E. Willcox, Youcef Mehellou

**Affiliations:** a School of Pharmacy and Pharmaceutical Sciences, Cardiff University Cardiff CF10 3NB UK MehellouY1@cardiff.ac.uk; b Institute of Immunology and Immunotherapy, University of Birmingham Birmingham B15 2TT UK b.willcox@bham.ac.uk; c Cancer Immunology and Immunotherapy Centre, University of Birmingham Birmingham B15 2TT UK; d School of Chemistry, University of Birmingham Birmingham B15 2TT UK; e Medicines Discovery Institute, Cardiff University Cardiff CF10 3AT UK

## Abstract

The phosphoantigen (*E*)-4-hydroxy-3-methyl-but-2-enyl pyrophosphate (HMBPP) is an established activator of Vγ9/Vδ2 T cells and stimulates downstream effector functions including cytotoxicity and cytokine production. In order to improve its drug-like properties, we herein report the design, synthesis, serum stability, *in vitro* metabolism, and biological evaluation of a new class of symmetrical phosphonodiamidate prodrugs of methylene and difluoromethylene monophosphonate derivatives of HMBPP. These prodrugs, termed phosphonodiamidate ProPAgens, were synthesized in good yields, exhibited excellent serum stability (>7 h), and their *in vitro* metabolism was shown to be initiated by carboxypeptidase Y. These phosphonodiamidate ProPAgens triggered potent activation of Vγ9/Vδ2 T cells, which translated into efficient Vγ9/Vδ2 T cell-mediated eradication of bladder cancer cells *in vitro*. Together, these findings showcase the potential of these phosphonodiamidate ProPAgens as Vγ9/Vδ2 T cell modulators that could be further developed as novel cancer immunotherapeutic agents.

## Abstract 2

Phosphonodiamidate prodrugs of the monophosphonate derivatives of HMBPP, a natural phosphoantigen, exhibit potent activation of Vγ9Vδ2 T cells resulting in the lysis of bladder cancer cells *in vitro*.

## Graphical abstract

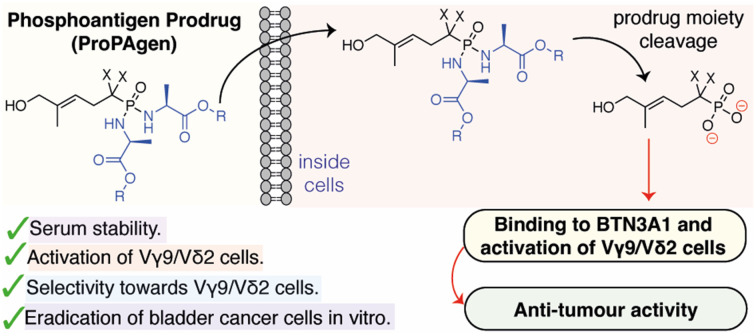

## 1. Introduction

Vγ9/Vδ2 T cells are cytotoxic lymphocytes that play a key role in immunosurveillance against malignancies and infection.^1,2^ Target recognition of these cells is dependent on their T cell receptor (TCR)^3^ and cell–cell contact, but unlike the widely studied αβ T cell compartment, it is major histocompatibility complex (MHC)-independent.^4,5^ Upon activation and recognition of target cells, Vγ9/Vδ2 T cells produce potent effector responses, including cytotoxicity and production of IFN-γ and TNFα.^6,7^

**Fig. 1:**
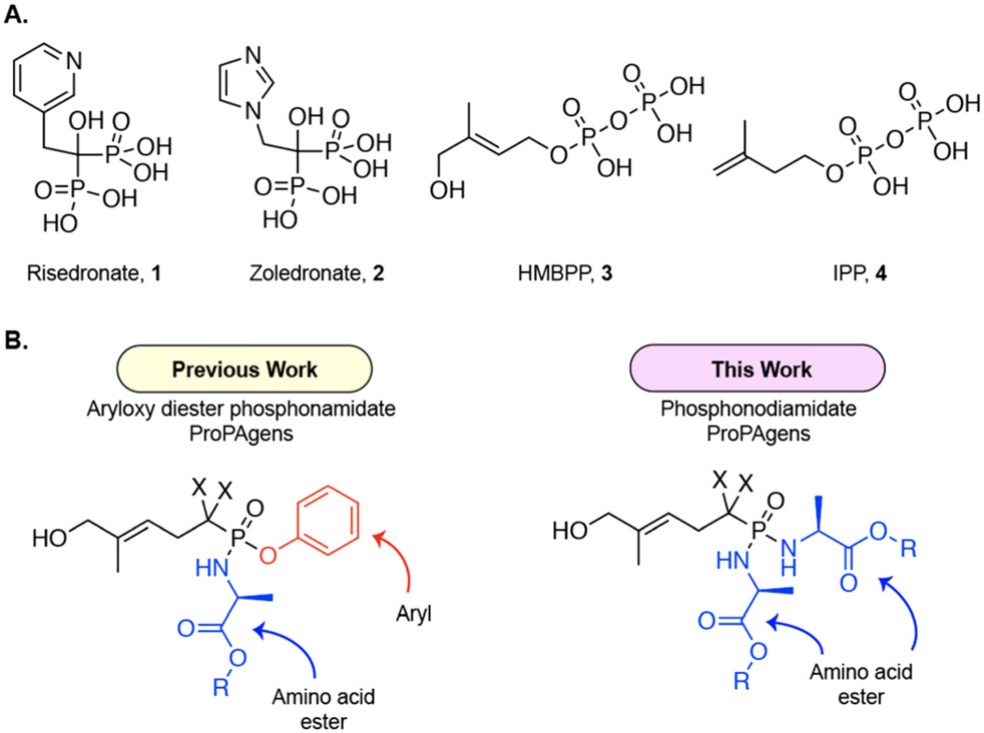
A. Chemical structures of key phosphate- and phosphonate-containing small molecule activators of Vγ9/Vδ2 T cells; HMBPP (EC_50_: 60–500 pM), IPP (EC_50_: 1–10 μM), risedronate (EC_50_: 0.08–5 μM), and zoledronate (EC_50_: 0.003–3.0 μM). B. General chemical structures of the monophosphonate derivative of the PAg HMBPP prodrugs, termed ProPAgens. Aryloxy diester phosphonamidate ProPAgens (left) and phosphonodiamidate ProPAgens (right). X = H or F.

Vγ9/Vδ2 T cells are activated by a handful of synthetic or naturally occurring phosphate- or phosphonate-containing small molecules. These include two aminobisphosphonate drugs, risedronate and zoledronate (Fig. 1A), which are currently used clinically to treat osteoporosis and some types of cancer.^8–10^ These agents inhibit isopentenyl pyrophosphate (IPP) catabolism *via* farnesyl diphosphate (FPP) synthase, which results in the intracellular accumulation of IPP, which ultimately results in Vγ9/Vδ2 T cell activation.^7,11,12^ In addition to these two synthetic compounds, two naturally occurring pyrophosphate-containing molecules have been identified as direct Vγ9/Vδ2 T cell activators. These are the microbially-derived phosphoantigen (PAg) (*E*)-4-hydroxy-3-methyl-but-2-enyl pyrophosphate (HMBPP) and the host-derived PAg isopentenyl pyrophosphate (IPP) itself (Fig. 1A).^5,13^ These activate Vγ9/Vδ2 T cells by binding to the intracellular domain of type-1 transmembrane protein butyrophilin 3A1 (BTN3A1) on target cells,^14^ which also co-express its second family member, butyrophilin 2A1 (BTN2A1).^15–17^ The PAgs binding to BTN3A1, leading to a conformational change in the cytoplasmic B30.2 domain of the BTN3A1 protein and promoting its association with BTN2A1,^18^ and this ultimately results in assembly of an activation complex recognized by the Vγ9/Vδ2 TCR.^15–17^ Although the exact mechanisms underpinning Vγ9/Vδ2 T cell PAg sensing are not completely understood, PAg binding to BTN3A1, by promoting its subsequent association with BTN2A1,^18^ likely catalyzes formation of a “composite ligand” on the target cell surface for the Vγ9/Vδ2 TCR complex, thereby triggering activation.^16,17^

With the aim of exploiting the immunotherapeutic potential of Vγ9/Vδ2 T cells, we focused our attention on improving the drug-like properties of HMBPP, the canonical microbially-derived PAg, which is a highly potent activator of Vγ9/Vδ2 T cells relative to the host-derived PAg IPP. Indeed, we previously reported that the aryloxy triester phosphoramidates of the monophosphate derivative of HMBPP (ProPAgens) exhibited potent activation of Vγ9/Vδ2 T cells, although these had limited serum stability (*t*_1/2_ < 30 min).^19^ Additionally, the aryloxy diester phosphonamidate^20,21^ and bis-pivaloyloxymethyl (bisPOM)^22^ prodrugs of the phosphatase-resistant monophosphonate derivatives of HMBPP displayed potent Vγ9/Vδ2 T cell activation and elicited their lysis of cancer cells *in vitro*. Subsequently, symmetrical diamidate prodrugs of HMBPP methylene monophosphonate were reported,^23^ but these did not include symmetrical diamidate prodrugs encompassing l-alanine, which has historically been shown to generate prodrugs associated with far superior pharmacological activity compared to other amino acids.^20,24^ Encouraged by the potency of HMBPP ProPAgens, in this work we report the design, synthesis, and biological evaluation of symmetrical l-alanine phosphonodiamidate prodrugs of the methylene and difluoromethylene monophosphonate derivatives of HMBPP (Fig. 1B). The purpose of using symmetrical phosphonodiamidate prodrugs was to remove the aryl group from these prodrugs, which could be associated with *in vivo* toxicity, simplify the synthesis and remove the chirality at the phosphorous centre. Critically, symmetrical phosphoramidate prodrugs of nucleotides showed promising pharmacological activity compared to McGuigan's highly successful aryloxy triester phosphoramidate (ProTide) prodrugs.^25,26^

## 2. Results and discussion

### 2.1. Design and synthesis of HMBP phosphonodiamidate ProPAgens

In the design of the phosphonodiamidate prodrugs, we elected to mask the phosphonate groups with two l-alanine containing amino acid esters. This choice was driven by the fact that phosphoramidate and phosphonamidate prodrugs containing l-alanine as the masking group have consistently shown superior pharmacological activity compared to other amino acid containing prodrugs.^24^ In terms of the ester groups, we employed four different groups; methyl (Me), isopropyl (*i*Pr), *tert*-butyl (*t*Bu), and benzyl (Bn) because they typically show varying biological activities that range from low (*t*Bu) to high (Bn).^19,24,27^

**Scheme 1:**
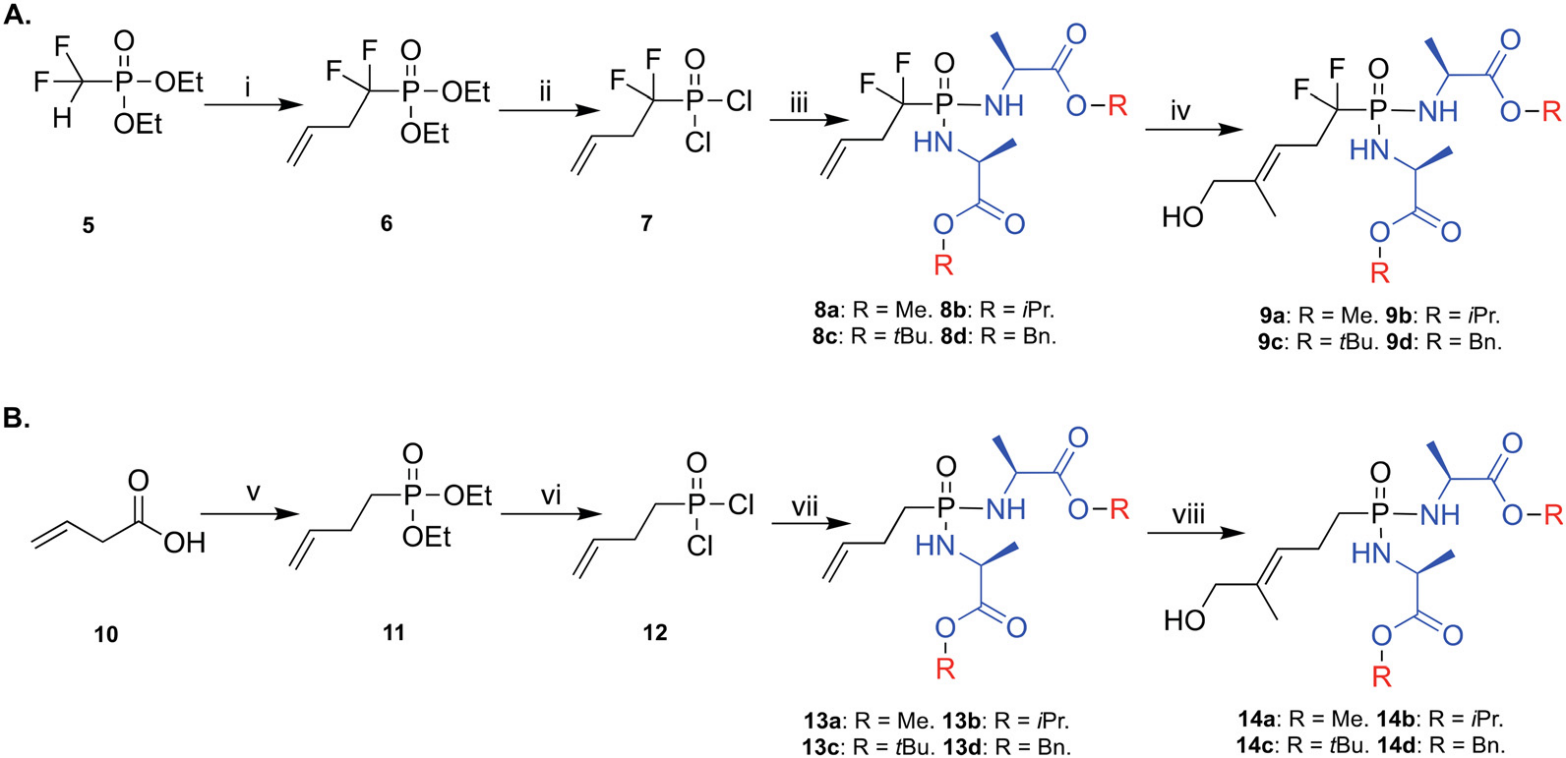
Synthesis of phosphonodiamidate prodrugs of methylene and difluoromethylene monophosphonate derivatives HMBPP. Reagents and conditions: A. (i) LDA, HMPA, THF, allyl bromide, −78 °C, yield 44%; (ii) TMSBr, DCM, 50 °C, N_2_, 3 h then (COCl)_2_, DMF cat, DCM, rt, 2 h; (iii) l-alanine ester hydrochloride, DCM, N_2_, −78 °C, TEA then rt, overnight, yield: 23–49%; (iv) 2-methyl-2-propenol, 1,4-benzoquinone, Hoveyda–Grubbs catalyst 2nd generation, DCM, 78 °C, yields: 38–67%. B. (v) (COCl)_2_ (solvent and reagent), N_2_, 0 °C to rt, DMF then (EtO)_3_P, 0 °C to rt, overnight, yield 51%; (vi), TMSBr, DCM, 50 °C, N_2_, 3 h then (COCl)_2_, DMF cat, DCM, rt, 2 h; (vii) l-alanine ester hydrochloride, DCM, N_2_, −78 °C, TEA then rt, overnight, yield: 23–44%; (viii) 2-methyl-2-propenol, 1,4-benzoquinone, Hoveyda–Grubbs catalyst 2nd generation, DCM, 78 °C, yields: 33–63%.

The synthesis of the prodrugs started by making the backbone of the two methylene and difluoromethylene monophosphonate derivatives of HMBPP, which was accomplished as we previously reported (Scheme 1).^20^ Briefly, for the difluoromethylene monophosphonate backbone, the synthesis started by reacting the commercially available α,α-difluorophosphonate 5 with allyl bromide in THF and in the presence of lithium diisopropylamine (LDA) and hexamethylphosphoramide (HMPA), as reported,^28,29^ to yield product 6 in 44% yield. Subsequently, compound 6 was treated with trimethylsilyl bromide (TMSBr) at room temperature to remove the ethoxy groups and generate the phosphonic acid derivative.^30^ This was followed by a chlorination reaction using oxalyl chloride in the presence of a catalytic amount of DMF to generate compound 7, which was used in the next reaction without purification. Next, compound 7 was treated with 2.5 equivalents of the appropriate amino acid ester in the presence of triethylamine and this generated the desired phosphonodiamidates 8a–d in good yields (23–49%). Finally, these compounds underwent Grubbs olefin metathesis^31^ with 2-methyl-2-propenol employing the Hoveyda–Grubbs second generation catalyst in the presence of 1,4-benzoquinone to prevent alkene isomerization.^32^ This gave the desired phosphonodiamidate ProPAgens 9a–d in good yields (38–67%). For ProPAgens 14a–d, their synthesis was achieved in the same manner as that used for making 8a–d, with the only exception being the preparation of compound 11. This was achieved by first reacting 3-butenoic acid (10) with oxalyl chloride in the presence of DMF to generate 3-butenoyl chloride, which was subsequently reacted with triethylphosphite ((EtO)_3_P) to yield compound 11 in a good yield (51%). The phosphonodiamidate ProPAgens 14a–d were obtained in good yields, 33–63%. The final HMBP phosphonodiamidate ProPAgens, 9a–d and 14a–d, were obtained in ≥95% purity (Fig. S1†).

### 2.2. Serum stability of HMBP phosphonodiamidate ProPAgens

**Fig. 2:**
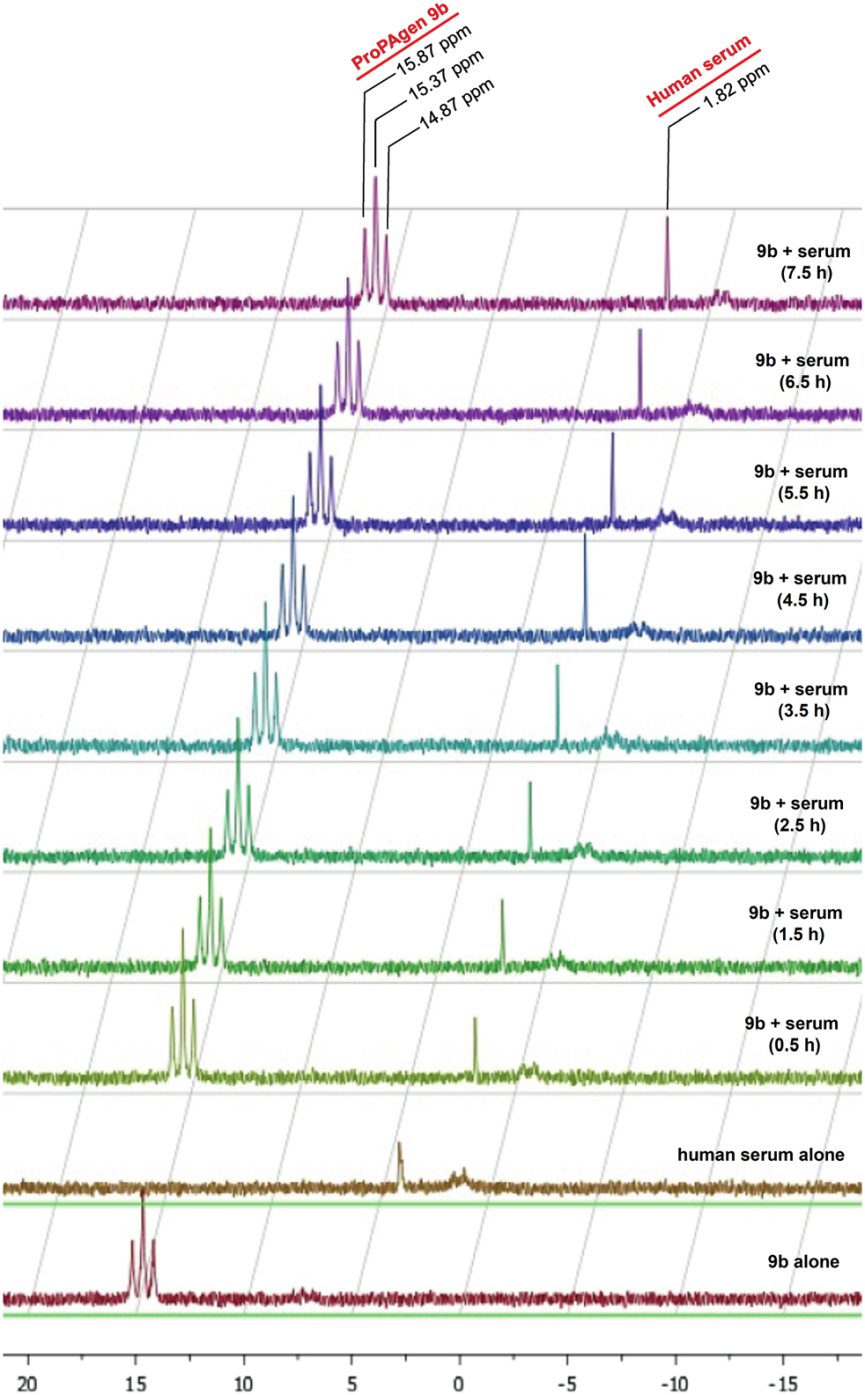
Stability of the HMBP phosphonodiamidate ProPAgen 9b in human serum at 37 °C for 7.5 h as monitored by ^31^P NMR. The assay sample contained 5 mg of ProPAgen 9b which was initially dissolved in 50 μL DMSO/150 μL D_2_O followed by the addition of 300 μL of human serum.

Upon synthesis, we first studied whether these phosphonodiamidate ProPAgens are stable in human serum. As a representative of this class of phosphonodiamidate ProPAgens in the serum stability studies, we chose ProPAgen 9b, which has an *i*Pr ester like the two FDA-approved phosphoramidate prodrugs, sofosbuvir and tenofovir alafenmide.^24,33^ Thus, ProPAgen 9b was incubated with human serum at 37 °C for 12 h and monitored the sample by ^31^P NMR, as we reported previously.^20^ As shown in Fig. 2, the ^31^P NMR spectra of ProPAgen 9b gave a triplet centred at *δ*_P_ = 15.37 ppm for its phosphorous coupled to two fluorine atoms. Notably, the human serum also showed a ^31^P-NMR peak at *δ*_P_ = 1.82. Following the incubation of the phosphonodiamidate ProPAgen 9b with human serum and monitoring of the sample by ^31^P-NMR, these original 9b^31^P-NMR peaks remained present through the 7.5 h of the study, and no new ^31^P-NMR peaks were observed. These data indicated excellent human serum stability of 9b (*t*_1/2_ > 7.5 h). This stability profile is in line with the aryloxy diester phosphoramidate prodrugs of these monophosphonates that we reported^20^ before and that of the analogous symmetrical nucleoside phosphorodiamidate prodrugs.^26^

### 2.3. *In vitro* metabolism of HMBP phosphonodiamidate ProPAgens

**Fig. 3:**
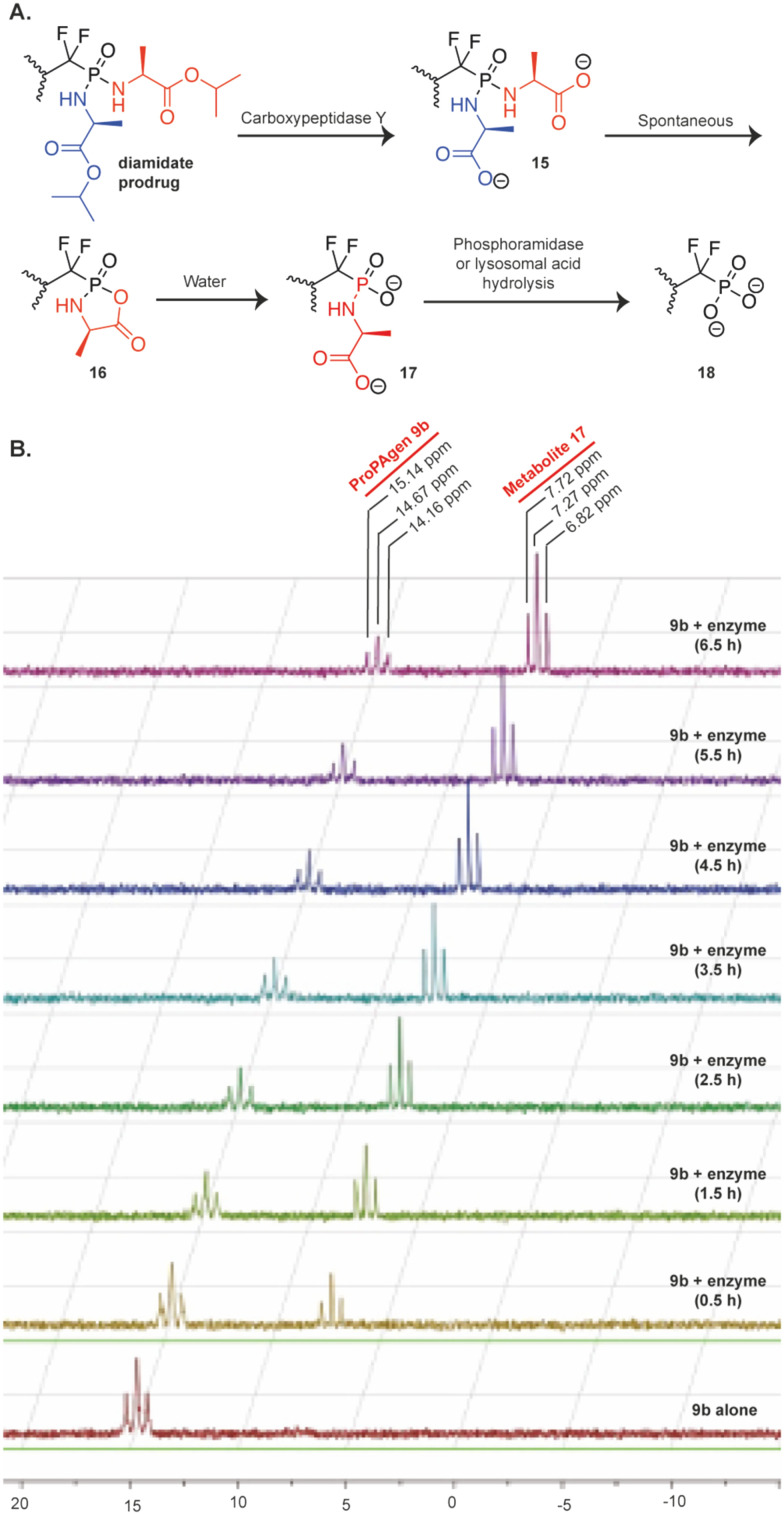
Metabolism of diamidate ProPAgens. A. Postulated mechanism of phosphonodiamidate prodrugs as suggested by McGuigan *et al.* B. *In vitro* carboxypeptidase Y-mediated breakdown of the phosphonodiamidate ProPAgen 9b. ^31^P-NMR spectrum of ProPAgen 9b alone and at different time points (as shown), following incubation with recombinant carboxypeptidase Y at 37 °C for 6.5 h.

Next, we studied the *in vitro* metabolism of these phosphonodiamidate ProPAgens. The reported metabolism of these phosphonodiamidate prodrugs is suggested to be initiated with an esterase, *e.g.*, carboxypeptidase Y, which removes the ester motif from the amino acid esters and frees the carboxylate groups (15, Fig. 3A).^26^ This is then followed by a spontaneous nucleophilic attack from one of the carboxylate groups onto the phosphorous center, which triggers leaving of the second amino acid and the generation of an unstable five membered ring (16, Fig. 3A).^26^ The next metabolism step involves a nucleophilic attack from a water molecule onto the phosphorous or carbonyl group to generate phosphoramidate (17, Fig. 3A).^26^ Finally, the cleavage of the P–N bond in metabolite 17 is achieved *via* the activity of phosphoramidase-type enzymes, *e.g.*, hint-1,^25^ or lysosomal acid hydrolysis^34,35^ releasing the unmasked monophosphonate species (18, Fig. 3A).

With this postulated mechanism of phosphonodiamidate prodrugs in mind, we subsequently incubated the phosphonodiamidate ProPAgen 9b with recombinant carboxypeptidase Y at 37 °C and monitored the reaction by ^31^P-NMR for 6.5 h. The results demonstrated that at *t* = 0 in the buffer of the reaction, ProPAgen 9b showed a triplet with ^31^P NMR peaks at *δ*_P_ = 14.16, 14.67 and 15.14 ppm as expected (Fig. 3B). Upon addition of carboxypeptidase Y and within 0.5 h, a new ^31^P NMR triplet (*δ*_P_ = 6.82, 7.27 and 7.72 ppm) appeared and became prominent as the assay proceeded while the original ^31^P NMR peaks (*δ*_P_ = 14.16, 14.67 and 15.14 ppm) corresponding to ProPAgen 9b reduced over time. However, these did not get consumed completely suggesting that ProPAgen 9b was not fully metabolised during the assay period of 6.5 h (Fig. 3B). Notably, the ^31^P NMR shift of the new triplet peaks that emerged corresponds to that of metabolite 17 (Fig. 3B), akin to what we observed for this metabolite (*δ*_P_ = 6.50, 6.90 and 7.20 ppm) previously.^20^

### 2.4. Vγ9/Vδ2 T cells activation and cancer cells lysis by HMBP phosphonodiamidate ProPAgens

**Fig. 4:**
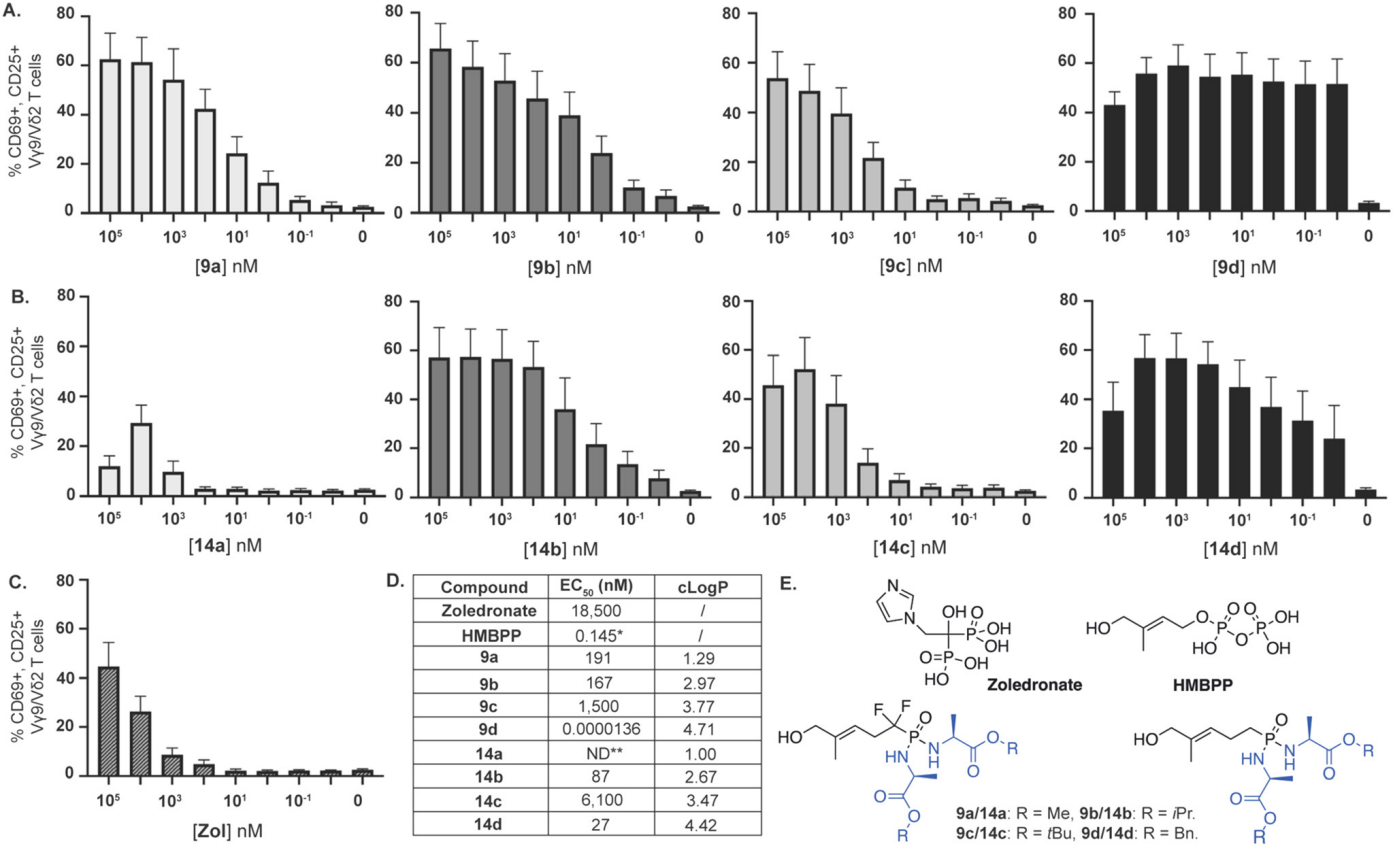
*In vitro* phosphonodiamidate ProPAgen-mediated activation of Vγ9/Vδ2 T cells, following overnight incubation with zoledronate, and HMBP ProPAgens 9a–d and 14a–d. Levels of activation are represented as % of Vγ9/Vδ2 T cells that are CD69+ CD25+. Data is shown as mean ± SE (*n* = 4). (A) Activation of Vγ9/Vδ2 stimulated by phosphonodiamidate ProPAgens 9a–d. (B) Activation of Vγ9/Vδ2 stimulated by ProPAgens 14a–d. (C) Activation of Vγ9Vδ2 stimulated by zoledronate (Zol). (D) EC_50_ values were calculated using GraphPad Prism v9 based on the results of the activation assay. Calculated log *P* (clog *P*) values were calculated using ChemDraw Professional 16.0. *EC_50_ value taken from Kadri *et al.*^20^ **ND: not determined. (E) Chemical structures of the compounds with EC_50_ values shown in D.

Subsequently, we studied the ability of these HMBP phosphonodiamidate ProPAgens to activate Vγ9/Vδ2 T cells and elicit their lysis of cancer cells *in vitro*. For this, peripheral blood mononuclear cells (PBMCs) derived from healthy donors and containing Vγ9/Vδ2 T cells were incubated with increasing concentrations of zoledronate or phosphonodiamidate HMBP ProPAgens 9a–d and 14a–d (up to 100 μM) (Fig. 4). Peripheral blood γδ T cells lack appreciable levels of surface CD69 or CD25 under steady-state conditions, but TCR stimulation upregulates both T cell activation markers.^6^ Thus, PAg-responsive Vγ9/Vδ2 T cells were then assessed for the upregulation of CD69 and CD25.

In the activation assay, zoledronate (Zol), a positive control, exhibited significant activation of Vγ9/Vδ2 T cells with an EC_50_ = of 18.5 μM, (Fig. 4C), broadly consistent with but marginally weaker than previous measurements in this identical assay system.^20^ As for the activation of Vγ9/Vδ2 T cells by the phosphonodiamidate ProPAgens (9a–d and 14a–d), these were initially tested using a concentration range from 0.1 nM to 100 μM (Fig. 4A and B). The results showed that these phosphonodiamidate ProPAgens exhibited varying levels of activation that range from super potent activation EC_50_ = 0.0000136 nM to 6.1 μM, and these were largely in line with the established structure–activity relationship (SAR) of aryloxy and diamidate phosphoramidate prodrugs.^24^ Indeed, in the fluorinated and non-fluorinated series, the phosphonodiamidate ProPAgens bearing a *tert*-butyl ester (9c and 14c) exhibited the least potent activation of Vγ9/Vδ2 T cells, EC_50_ = 1.5 and 6.1 μM, respectively (Fig. 4A and B). This was followed by the methyl ester phosphonodiamidate prodrugs where 9a exhibited good potency, EC_50_ = 191 nM, though we were unable to obtain an accurate potency level of 14a. Across the two series of the phosphonodiamidate ProPAgens, those bearing an isopropyl or benzyl ester exhibited the most potent activation of Vγ9/Vδ2 T cells *in vitro*. Indeed, phosphonodiamidate ProPAgens 9b and 14b showed good activation of Vγ9/Vδ2 T cells, EC_50_ = 167 and 87 nM, respectively. However, the phosphonodiamidate ProPAgens, 9d and 14d exhibited the most potent activation of Vγ9/Vδ2 T cells and ProPAgen 9d was the most potent in terms of Vγ9/Vδ2 T cell activation across the eight ProPAgens studied in this work, EC_50_ = 13.6 fM (Fig. 4A and C). In order to determine the accurate potencies (EC_50_) for the two benzyl phosphonodiamidate prodrugs 9d and 14d, we performed the activation assay with a concentration range of 10 aM to 100 μM (Fig. S2†). Notably, the extremely high Vγ9/Vδ2 T cell activation potency of the phosphonodiamidate ProPAgen 9d is comparable to its corresponding aryloxy diester phosphoramidate derivative, EC_50_ = 9.2 fM, which we reported on previously.^20^ Notably, at the highest concentration studied (100 μM), the activation of Vγ9/Vδ2 T cells by the phosphonodiamidate ProPAgens 9d and 14d was less than that achieved with 10 μM (Fig. 4A and B). This could be explained by negative feedback mechanisms induced by antigen overstimulation that lead to downregulation of the TCR, subsequently resulting in lower expression of the CD25 activation marker.^36^ This was also observed with the aryloxy diester phosphoramidate prodrug of HMBP methylene and difluoromethylene monophosphonates.^20^

The SAR observed in this work whereby the phosphonodiamidate prodrugs bearing benzyl ester were the most active is similar to that observed previously from studies on McGuigan's phosphoramidate and phosphorodiamidate prodrugs.^20,37–40^ Although in this work, the benzyl ester bearing phosphonodiamidate ProPAgen 9d exhibited potent Vγ9/Vδ2 T cell activation, its non-fluorinated ProPAgen derivative, 14d, was significantly less active than expected, with the activation potency of ProPAgens 9d and 14d predicted to be rather similar due to their structural similarity. However, considering the chemical structures of ProPAgens 9d and 14d, it is apparent that the phosphonate centre in ProPAgen 9d is comparatively more activated than that of ProPAgen 14d due to the presence of the electron-withdrawing difluoro atoms. This makes the phosphorous centre in ProPAgen 9d more electron-deficient than that of ProPAgen 14d. As a result, upon the ester cleavage of these ProPAgens, as shown in Fig. 3, the nucleophilic attack from the carboxylate group onto the phosphorous centre proceeds faster for ProPAgen 9d compared to ProPAgen 14d. Hence, the metabolism of ProPAgen 9d is likely to proceed faster than that of ProPAgen 14d, and consequently the metabolite, which activates Vγ9/Vδ2 T cells, is generated more quickly as compared to ProPAgen 14d metabolism. It is also worth noting that the cellular activity of this type of phosphonodiamidate prodrug is often determined following 72 h incubation,^25,26^ while in this study and for the activation of Vγ9/Vδ2 T cells, we incubated the ProPAgens overnight, *ca.* 12 h. Hence, one could envisage that in the overnight incubation in this work, and given the metabolism of ProPAgen 9d being likely to proceed faster than that of ProPAgen 14d, ProPAgen 9d was more efficiently metabolised in this assay period to release the active metabolite and induce Vγ9/Vδ2 T cell activation compared to ProPAgen 14d. Despite this hypothesis, further mechanistic and metabolic studies are needed to elucidate the reasons for the significant differences in activation potencies between the diamidate ProPAgens 9d and 14d. In the meantime, and to ensure the samples used were not compromised upon dissolution and storage, we analysed the samples again after the assay by mass spectrometry and HPLC, confirming their purity (Fig. S3†).

After establishing the ability of HMBP phosphonodiamidate ProPAgens to activate Vγ9/Vδ2 T cells, we subsequently studied their specificity towards the activation of Vγ9/Vδ2 T cells. Indeed, we assessed the activation of CD8^+^ αβ T cells, which are not activated by PAgs, but by peptides.^41^ As expected, our data confirmed that these phosphonodiamidate ProPAgens did not induce any activation of αβ T cells (Fig. S4†). The specificity of these HMBP phosphonodiamidate ProPAgens is, therefore, in line with that observed with the aryloxy diester phosphonamidate ProPAgens of HMBP.^20^

**Fig. 5:**
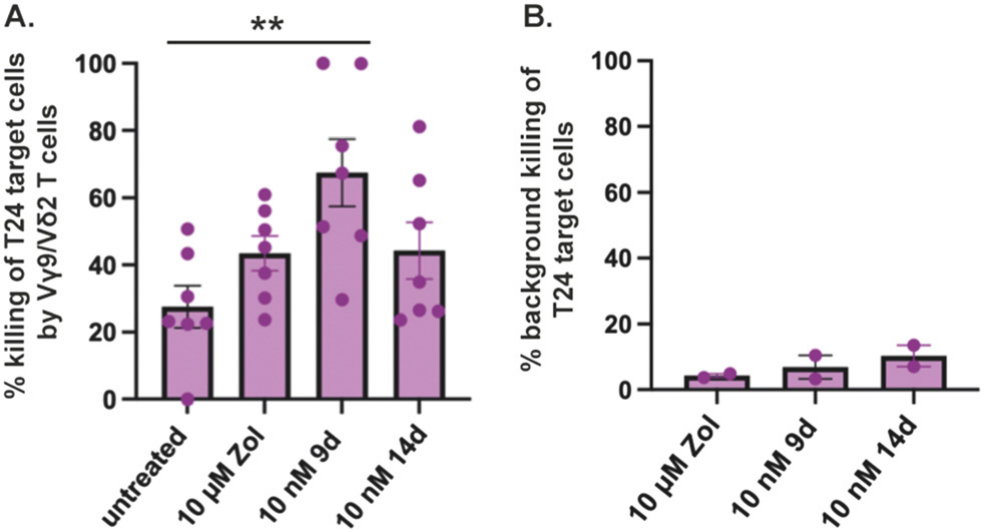
Cytotoxicity of Vγ9/Vδ2 T cells toward the phosphonodiamidate ProPAgen-treated T24 cells. % killing of T24 cells was calculated with this formula: [(experimental release − spontaneous release)/(maximal release − spontaneous release)] × 100. The definitions of each of these variables are provided by the assay manufacturer (DELFIA). A. Cytotoxicity of phosphonodiamidate ProPAgens 9d and 14d (used at 10 nM) compared to the 10 μM zoledronate -induced effect. B. As in A, but without Vγ9/Vδ2 T cells. Data is shown as mean ± SE (*n* = 7). Statistical analysis was performed using one-way ANOVA and Tukey's multiple comparisons test on GraphPad Prism v9. **p < 0.0063.

Encouraged by the potency and specificity of our HMBP phosphonodiamidate ProPAgens, especially 9d and 14d, we subsequently studied their ability to sensitize the urinary bladder carcinoma cell line T24 for targeted killing by *in vitro* expanded Vγ9/Vδ2 T cells (Fig. 5). In brief, T24 cells were incubated in PBS containing 10 μM zoledronate or the indicated HMBP phosphonodiamidate ProPAgens for a period of 2 h. A positive control for cell death (target cells incubated with 10% v/v DELFIA lysis buffer) and media-only treated control (no drug) was also included. The cells were then washed and cocultured with *ex vivo* expanded Vγ9/Vδ2 T cells for 1 h at 80 : 1 effector : target ratio, and the level of killing of T24 cells was then measured *via* time-resolved fluorescence. The data show that the sensitizing effects of 10 nM phosphorodiamidate ProPAgens 9d and 14d was evidently much more potent in comparison to 10 μM zoledronate (Fig. 5A).

## 3. Conclusions

Inspired by the anti-tumour potential of HMBPP prodrugs, we herein present a new and novel series of phosphonodiamidate prodrugs of the PAg HMBP. In particular, we discussed in this work the design, synthesis, and biological evaluation of methylene and difluoromethylene monophosphonate derivatives of the PAg HMBP. These prodrugs exhibited excellent stability in human serum and elicited potent activation of Vγ9/Vδ2 T cells, which triggered potent *in vitro* killing of the urinary bladder carcinoma cell line T24. The stability, *in vitro* activation and efficacy of these phosphonodiamidate prodrugs was comparable to that we previously observed with the aryloxy diester phosphonamidates^20^ and the mixed aryl prodrugs of this PAg.^22^

The observed superior activation of Vγ9/Vδ2 T cells by the HMBP phosphonodiamidate ProPAgens compared to zoledronate and HMBPP^20^ is due to their improved uptake into cells as they are less polar. Indeed, it is well established in the literature that the application of the aryloxy diester/triester phosphor(n)amidate or phosphor(n)odiamidate prodrug technology leads to the generation of more lipophilic prodrugs compared to the parent compound and these prodrugs access cells *via* passive diffusion and release the active metabolite intracellularly.^24,25,39,40^ For our phosphonodiamidate ProPAgens of HMBP, their ability to activate Vγ9/Vδ2 T cells in PBMC assays is due to their uptake into cells and metabolism by esterases and phosphoramidase-type enzymes or lysosomal acid hydrolysis to release HMBP. This is a moderately potent PAg (EC_50_ = 4 μM)^42^ and which is likely to be further phosphorylated one more time to generate a phosphonate derivative of HMBPP that is a highly potent activator (EC_50_ = 0.00051 μM)^42^ of Vγ9/Vδ2 T cells. Once the PAg is released inside cells, it then binds to the intracellular domain of the ubiquitously expressed BTN3A1 transmembrane receptor, leading to a conformational change, promoting its association with BTN2A1,^18^ and leading to assembly of an activation complex that is ultimately recognised by the Vγ9/Vδ2 TCR.^16,17^

Although HMBP phosphonodiamidate ProPAgens induced significant Vγ9/Vδ2 cytotoxicity towards the urinary bladder carcinoma cell line T24, the ubiquitous expression of the BTN3A1 receptor, the molecular target of PAgs, in non-cancer cells suggests that these ProPAgens may be cytotoxic towards non-cancer cells. However, previous *in vivo* studies using a phosphoantigen mimic termed bromohydrin pyrophosphate (BrHPP),^43^ which activates Vγ9/Vδ2 T cells^43^*via* the involvement of BTN3A1,^44^ found this compound to be safe and well tolerated in cancer patients when used in combination with a low dose of IL-2.^45,46^ While further investigation of cancer cell selectivity for our HMBP ProPAgens is required, these previous findings establish a precedent that at least some phosphoantigens can achieve acceptable safety profiles in patients when used in combination with IL-2.

Overall, the HMBP phosphonodiamidate ProPAgens discussed in this work represent a new class of small molecule activators of Vγ9/Vδ2 T cells that warrant further *in vivo* safety and efficacy studies, and future development as new immunotherapeutics for treating challenging cancers and infections that can be targeted by Vγ9/Vδ2 T cell responses. Although these phosphonodiamidate ProPAgens could be explored as a monotherapy regimen, they could also be studied as part or subsequent to a clinical regimen to expand γδ T cells *in vivo* or, alternatively, they could be administered to patients receiving adoptive cell therapy with *ex vivo*-expanded γδ T cells, to directly augment Vγ9/Vδ2 T cell-mediated antitumor activity.

## Experimental section

### General information

All reagents and solvents were of general purpose or analytical grade and were purchased from Sigma-Aldrich Ltd., Fisher Scientific, Fluorochem, or Acros. ^31^P, ^1^H, ^19^F and ^13^C NMR data were recorded on a Bruker AVANCE DPX500 spectrometer operating at 202, 500, and 125 MHz, respectively. Chemical shifts (*δ*) are quoted in ppm, and *J* values are quoted in Hz. In reporting spectral data, the following abbreviations were used: s (singlet), d (doublet), t (triplet), q (quartet), dd (doublet of doublets), td (triplet of doublets), and m (multiplet). All of the reactions were carried out under a nitrogen atmosphere and were monitored using analytical thin layer chromatography on precoated silica plates (Kiesel gel 60 F254, BDH). Compounds were visualized by illumination under UV light (254 nm) or by the use of KMnO_4_ stain followed by heating. Flash column chromatography was performed with silica gel 60 (230–400 mesh) (Merck). HPLC was carried out on a SHIMADZU Prominence-i quaternary low-pressure gradient pump with a Prominence-i UV detector (190 to 700 nm). All solvents for HPLC were HPLC grade purchased from Fisher Scientific. HPLC data analysis was performed using the SHIMADZU Lab solutions software package. The purity of the tested ProPAgens was determined by HPLC, and they were all of ≥95% purity.

### Diethyl(1,1-difluorobut-3-en-1-yl)phosphonate (6)

To a solution of lithium diisopropylamine (LDA) (1.0 M in hexane/THF, 7.97 mL, 1 eq., 7.97 mmol) and hexamethylphosphoramide (HMPA) (1.38 mL, 1 eq., 7.97 mmol) in 5 mL THF at −78 °C was added a cooled solution of diethyl α,α-difluorophosphonate (1.7 mL, 1 eq., 7.97 mmol) in 3 mL THF. After stirring for 2 min, allyl bromide (5 mL, 1.2 eq., 9.56 mmol) was added quickly under fierce stirring. After 10 min, the reaction was quenched by NH_4_Cl and extracted by diethyl ether (10 mL) and ethyl acetate (2 × 20 mL). The combined organic phase was dried over MgSO_4_ and concentrated under reduced pressure. The crude was separated by flash column chromatography (EtOAc : hexane 4 : 6). Yield: 793 mg (44%). ^1^H NMR (500 MHz, CDCl_3_): 5.83–5.91 (m, 1H, CH=CH_2_), 5.31 (s, 1H, CH=CH_2_, *trans*), 5.29 (d, *J* = 5.1 Hz, 1H, CH=CH_2_, *cis*), 4.26–4.32 (m, 4H, 2 × CH_2_CH_3_), 2.80–2.91 (m, 2H, CH_2_CH=CH_2_), 1.40 (t, J = 7.1 Hz, 6H, 2 × CH_2_CH_3_). ^13^C NMR (125 MHz, CDCl_3_): 127.0, 126.9, 121.34, 64.42 (d, *J* = 6.9 Hz), 38.49–38.96 (m), 16.39 (d, *J* = 5.5 Hz).^31^P NMR (202 MHz, CDCl_3_): 6.94 (t, *J* = 107.4 Hz). ^19^F NMR (470 MHz, CDCl_3_): −111.23 (d, *J* = 108.4 Hz).

### Diethyl but-3-en-1-ylphosphonate (11)

This product was synthesized over two steps. First, 3-butenoic acid (1.5 mL, 1 eq., 6 mmol) and oxalyl chloride (1 mL, 2 eq., 12 mmol) were added to a round bottom flask under nitrogen inert atmosphere. The mixture was cooled down to 0 °C and three drops of DMF were added to catalyze the reaction. The mixture was allowed to warm up to room temperature and monitored by TLC. When starting materials spot disappeared, the excess oxalyl chloride was removed under reduced pressure and the crude, 3-butenoyl chloride, was used for the next step without purification. In the second step, a dry round bottom flask was charged with the crude 3-butenoyl chloride from the previous step and cooled down to 0 °C. Then, (EtO)_3_P was added dropwise. The mixture was allowed to warm up to room temperature and stirred overnight. After removing solvents under reduced pressure, the crude was purified by column chromatography (EtOAc : hexane 1 : 1). Yield: 626 mg (51%). ^1^H NMR (500 MHz, CDCl_3_): 5.92–6.03 (m, 1H, CH=CH_2_), 5.26–5.31 (m, 2H, CH=CH_2_), 4.08–4.22 (m, 4H, 2 × CH_2_CH_3_), 3.31 (dt, *J* = 7.0, 1.43, 2H, COCH_2_), 1.35 (t, *J* = 7.1, 6H, 2 × CH_2_CH_3_). ^13^C NMR (125 MHz, CDCl_3_): 173.90, 134.92, 119.53, 62.84, 38.54, 16.20. ^31^P NMR (202 MHz, CDCl_3_): 9.07 (s).

### General procedure for synthesizing 7 and 12

These compounds were synthesized over two steps. First, compounds 6 or 11 (1 eq.) was dissolved in 5 mL DCM and TMSBr (10 eq.) was added under nitrogen inert atmosphere. The reaction was refluxed at 50 °C for 3 h, and then the solvents and excess TMSBr were evaporated under reduced pressure. The crude product was then chlorinated by dissolving in 10 mL DCM and three drops of DMF were added as a catalyst under nitrogen inert atmosphere. After that, oxalyl chloride (10 eq.) was added dropwise. The reaction was stirred at room temperature for 2 h and solvents and excess oxalyl chloride were removed under reduced pressure. The crude product, 7 or 12, was used in the next step without further purification.

### General procedure for synthesizing 8a–d and 13a–d

Compounds 7 or 12 (1 eq.) were dissolved in 10 mL DCM under nitrogen inert atmosphere along with the appropriate l-alanine ester hydrochloride (2.5 eq.). The mixtures were then cooled down to −78 °C and TEA (4 eq.) was added dropwise. The reactions were allowed to warm up to room temperature and stirred overnight. The solvent was removed under reduced pressure and the crude was dissolved in EtOAc. Upon filtration, the filtrate was concentrated and the crude product was purified by flash column chromatography (EtOAc : hexane 1 : 1) to give the desired product.

### Dimethyl 2,2′-(((1,1-difluorobut-3-en-1-yl)phosphoryl)bis(azanediyl))(2*S*,2′*S*)-dipropionate (8a)

Yield: 144 mg (36%). ^1^H NMR (500 MHz, CDCl_3_): 5.81–5.89 (m, 1H, CH=CH_2_), 5.26–5.31 (m, 2H, CH=CH_2_), 4.05–4.15 (m, 2H, 2 × NHCH), 3.75 (d, *J* = 4.2 Hz, 6H, 2 × OCH_3_), 3.53 (t, *J* = 10.6 Hz, 1H, NH), 3.34 (t, *J* = 11.7 Hz, 1H, NH), 2.85 (tt, *J* = 6.4 Hz, 29.09 Hz, 2H, CH_2_CF_2_), 1.44 (t, *J* = 7.2 Hz, 6H, 2 × NHCHCH_3_). ^13^C NMR (125 MHz, CDCl_3_): 176.6, 127.4, 121.5, 52.6, 48.8, 38.1, 21.6. ^31^P NMR (202 MHz, CDCl_3_): 12.82 (t, *J* = 95.6 Hz). ^19^F NMR (470 MHz, CDCl_3_): −110.08 (d, *J* = 96.3 Hz), −110.66 (d, *J* = 54.1 Hz), −110.86 (d, *J* = 54.2 Hz), −111.44 (d, *J* = 96.1 Hz).

### Diisopropyl 2,2′-(((1,1-difluorobut-3-en-1-yl)phosphoryl)bis(azanediyl))(2*S*,2′*S*)-dipropionate (8b)

Yield: 209.4 mg (49%). ^1^H NMR (500 MHz, CDCl_3_): 5.81–5.89 (m, 1H, CH=CH_2_), 5.27–5.30 (m, 2H, CH=CH_2_), 5.01–5.06 (m, 2H, OCH(CH_3_)_2_), 3.99–4.12 (m, 2H, 2 × NHCH), 3.57 (t, *J* = 10.6 Hz, 1H, NH), 3.34 (t, *J* = 11.5 Hz, 1H, NH), 2.85 (tt, *J* = 6.2 Hz, 28.97 Hz, 2H, CH_2_CF_2_), 1.40–1.44 (m, 6H, 2 × NHCHCH_3_), 1.24–1.28 (m, 12H, OCH(CH_3_)_2_). ^13^C NMR (125 MHz, CDCl_3_): 173.3, 127.5, 121.4, 69.2, 48.9, 37.9, 21.6, 21.9. ^31^P NMR (202 MHz, CDCl_3_): 12.78 (t, *J* = 95.4 Hz). ^19^F NMR (470 MHz, CDCl_3_): −110.18 (d, *J* = −110.2 Hz), −110.74 (d, *J* = 31.2 Hz), −110.94 (d, *J* = 30.2 Hz), −111.49 (d, *J* = 94.8 Hz).

### Di-*tert*-butyl 2,2′-(((1,1-difluorobut-3-en-1-yl)phosphoryl)bis(azanediyl))(2*S*,2′*S*)-dipropionate (8c)

Yield: 113 mg (25%). ^1^H NMR (500 MHz, CDCl_3_): 5.81–5.89 (m, 1H, CH=CH_2_), 5.26–5.29 (m, 2H, CH=CH_2_), 3.92–4.01 (m, 2H, 2 × NHCH), 3.54 (t, *J* = 10.7 Hz, 1H, NH), 3.31 (t, *J* = 11.5 Hz, 1H, NH), 2.84 (tt, *J* = 6.0 Hz, 29.49 Hz, 2H, CH_2_CF_2_), 1.46 (d, *J* = 5.0 Hz, 18H, 2 × OC(CH_3_)_3_), 1.39 (t, *J* = 7.5 Hz, 6H, 2 × NHCHCH_3_). ^13^C NMR (125 MHz, CDCl_3_): 173.0, 127.5, 121.3, 82.0, 49.4, 37.9, 27.9, 21.7. ^31^P NMR (202 MHz, CDCl_3_): 12.80 (t, *J* = 95.0 Hz). ^19^F NMR (470 MHz, CDCl_3_): −110.35 (d, *J* = 94.5 Hz), −110.89 (d, *J* = 11.6 Hz), −111.09 (d, *J* = 12.0 Hz), −111.62 (d, *J* = 96.4 Hz).

### Dibenzyl 2,2′-(((1,1-difluorobut-3-en-1-yl)phosphoryl)bis(azanediyl))(2*S*,2′*S*)-dipropionate (8d)

Yield: 122 mg (23%). ^1^H NMR (500 MHz, CDCl_3_): 7.30–7.37 (m, 10H, Ph), 5.77–5.82 (m, 1H, CH=CH_2_), 5.22–5.27 (m, 2H, CH=CH_2_), 4.11–4.16 (m, 2H, 2 × NHCH), 3.52 (t, *J* = 10.6 Hz, 1H, NH), 3.33 (t, *J* = 11.6 Hz, 1H, NH), 2.82 (tt, *J* = 6.4 Hz, 29.45 Hz, 2H, CH_2_CF_2_), 1.44 (d, *J* = 7.1 Hz, 3H, NHCHCH_3_), 1.36 (d, *J* = 7.1 Hz, 3H, NHCHCH_3_). ^13^C NMR (125 MHz, CDCl_3_): 176.6, 135.0, 134.6, 128.7, 128.6, 128.5, 128.4, 128.3, 128.2, 127.4, 121.5, 67.3, 48.7, 37.9, 21.3. ^31^P NMR (202 MHz, CDCl_3_): 12.76 (t, *J* = 95.6 Hz). ^19^F NMR (470 MHz, CDCl_3_): −109.99 (d, *J* = 96.3 Hz), −110.56 (d, *J* = 39.8 Hz), −110.76 (d, *J* = 39.4 Hz), −111.32 (d, *J* = 94.8 Hz).

### Dimethyl 2,2′-((but-3-en-1-ylphosphoryl)bis(azanediyl))(2*S*,2′*S*)-dipropionate (13a)

Yield: 424 mg (44%). ^1^H NMR (500 MHz, CDCl_3_): 5.86 (m, 1H, CH_2_=CH–), 5.07 (m, 2H, CH_2_=CH–), 4.03 (m, 2H, 2 × CHNH), 3.74 (d, *J* = 2.3 Hz, 6H, OCH_3_), 3.03, 2.93 (m, 2 × 1H, 2 × NH), 2.36 (m, 2H, PCH_2_CH_2_), 1.81 (m, 2H, PCH_2_CH_2_), 1.38 (d, *J* = 7.2 Hz, 6H, 2 × NHCHCH_3_). ^13^C NMR (125 MHz, CDCl_3_): 174.5, 156.25, 137.55, 115.47, 52.36, 48.81, 48.42, 28.94, 28.04, 26.86, 21.56, 18.99. ^31^P NMR (202 MHz, CDCl_3_): 28.78 (s).

### Diisopropyl 2,2′-((but-3-en-1-ylphosphoryl)bis(azanediyl))(2*S*,2′*S*)-dipropionate (13b)

Yield: 300 mg (26.5%). ^1^H NMR (500 MHz, CDCl_3_): 5.86 (m, 1H, CH_2_=CH–), 5.03 (m, 4H, CH_2_=CH–, 2 × CH(CH_*3*_)_2_), 3.96 (m, 2H, 2 × CHNH), 3.07, 2.98 (m, 2 × 1H, 2 × NH), 2.36 (m, 2H, PCH_2_CH_2_), 1.80 (m, 2H, PCH_2_CH_2_), 1.37 (m, 6H, 2 × NHCHCH_3_), 1.25 (m, 12H, OCH(CH_3_)_2_). ^13^C NMR (125 MHz, CDCl_3_): 174.27, 137.49, 115.36, 68.95, 49.00, 48.63, 29.05, 28.15, 26.88, 21.69, 19.16. ^31^P NMR (202 MHz, CDCl_3_): 28.59 (s).

### Di-*tert*-butyl 2,2′-((but-3-en-1-ylphosphoryl)bis(azanediyl))(2*S*,2′*S*)-dipropionate (13c)

Yield: 284 mg (23%). ^1^H NMR (500 MHz, CDCl_3_): 5.84 (m, 1H, CH_2_=CH–), 5.06 (m, 2H, CH_2_=CH–), 3.90 (m, 2H, 2 × CHNH), 3.03, 2.91 (m, 2 × 1H, 2 × NH), 2.35 (m, 2H, PCH_2_CH_2_), 1.78 (m, 2H, PCH_2_CH_2_), 1.46 (m, 18H, 2 × OC(CH_3_)_3_), 1.36 (m, 6H, 2 × NHCHCH_3_). ^13^C NMR (125 MHz, CDCl_3_): 173.8, 137.56, 115.24, 81.63, 49.26, 29.11, 28.22, 27.97, 26.89, 21.76. ^31^P NMR (202 MHz, CDCl_3_): 28.53 (s).

### Dibenzyl 2,2′-((but-3-en-1-ylphosphoryl)bis(azanediyl))(2*S*,2′*S*)-dipropionate (13d)

Yield: 400 mg (28%). 1H NMR (500 MHz, CDCl_3_): 7.35 (m, 10H, Ph), 5.79 (m, 1H, CH_2_=CH–), 5.15 (m, 4H, 2 × OCH_2_C_6_H_5_), 5.01 (m, 2H, CH_2_=CH–), 4.05 (m, 2H, 2 × CHNH), 3.00, 2.93 (m, 2 × 1H, 2 × NH), 2.31 (m, 2H, PCH_2_CH_2_), 1.76 (m, 2H, PCH_2_CH_2_), 1.42 (m, 6H, 2 × NHCHCH_3_). ^13^C NMR (125 MHz, CDCl_3_): 174.53, 137.54, 128.64, 128.45, 128.24, 115.44, 67.06, 48.76, 46.78, 28.98, 28.08, 26.84, 21.41, 8.63. ^31^P NMR (202 MHz, CDCl_3_): 28.71 (s).

### General procedure for synthesizing 9a–d and 14a–d

Compounds 8a–d and 13a–d (1 eq., 0.335 mmol) were dissolved in 10 mL DCM under nitrogen inert atmosphere. 2-Methyl-2-propen-1-ol (0.056 mL, 2 eq., 0.67 mmol), 1,4-benzoquinone (3.6 mg, 10% mol, 0.0335 mmol) and Hoveyda–Grubbs catalyst 2nd generation (5.22 mg added three times at 0, 3, 6 h (15.7 mg in total), 7.5% mol, 0.025 mmol) were added. The mixture was refluxed at 45 °C for 18 h and then cooled to room temperature. Activated charcoal was added and stirred for 1 h to absorb inorganic Ru from the catalyst. After that, the mixture was filtered through celite, concentrated under reduced pressure and purified by flash column chromatography (EtOAc : hexane, gradient from 20 : 80 to 100 : 0). Geometric configuration confirmed by NOESY.

### Dimethyl 2,2′-((((*E*)-1,1-difluoro-5-hydroxy-4-methylpent-3-en-1-yl)phosphoryl)bis(azanediyl))(2*S*,2′*S*)-dipropionate (9a)

Yield: 62 mg (38%). ^1^H NMR (500 MHz, CDCl_3_): 5.53 (m, 1H, CH_2_CH=CCH_3_CH_2_OH), 4.11 (m, 2H, 2 × NHCH), 4.04 (s, 2H, CH_2_OH), 3.76 (s, 6H, 2 × OCH_3_), 3.62(m, 1H, NHCH), 3.42 (m, 1H, NHCH), 2.91 (m, 2H, CF_2_CH_2_), 1.72 (s, 3H, CH=CCH_3_CH_2_OH), 1.44 (t, *J* = 6.9 Hz, 6H, 2 × NHCHCH_3_). ^13^C NMR (125 MHz, CDCl_3_): 176.58, 142.01, 113.34, 67.95, 52.69, 48.78, 32.56, 21.77, 21.41, 14.00. ^31^P NMR (202 MHz, CDCl_3_): 13.31 (t, *J* = 96.83 Hz). ^19^F NMR (470 MHz, CDCl_3_): −108.32 (d, *J* = 96.7 Hz), −108.91 (d, *J* = 66.5 Hz), −109.11 (d, *J* = 66.4 Hz), −109.70 (d, *J* = 96.5 Hz). HRMS (ES+, *m*/*z*): calcd for (M + Na)^+^ C_14_H_25_F_2_N_2_O_6_PNa, 409.1317; found, 409.1316. HPLC (reverse-phase) 0.5 mL min^−1^ MeOH/H_2_O 70 : 30 in 12 min, *λ* = 210 nm, *R*_t_ = 5.82 min (100%).

### Diisopropyl 2,2′-((((*E*)-1,1-difluoro-5-hydroxy-4-methylpent-3-en-1-yl)phosphoryl)bis(azanediyl))(2*S*,2′*S*)-dipropionate (9b)

Yield: 174 mg (52%). ^1^H NMR (500 MHz, CDCl_3_): 5.54 (m, 1H, CH=C), 5.04 (m, 2H, 2 × OCHCH_3_), 4.05 (m, 4H, CH_2_OH, 2 × CHNH), 3.57, 3.32 (m, 2 × 1H, 2 × NH), 2.89 (m, 2H, POCF_2_CH_2_), 2.30 (s, 1H, OH), 1.73 (s, 3H, CH_3_(CH_2_OH)C=CH), 1.42 (t, *J* = 6.7 Hz, 6H, 2 × NHCHCH_3_), 1.26 (m, 12H, OCH(CH_3_)_2_). ^13^C NMR (125 MHz, CDCl_3_): 173.6, 142.2, 113.5, 69.5, 68.0, 49.0, 32.7, 22.0, 21.6, 21.4, 14.0. ^31^P NMR (202 MHz, CDCl_3_): 13.18 (t, *J* = 96.6 Hz). ^19^F NMR (470 MHz, CDCl_3_): −108.85 (d, *J* = 12.0 Hz), −109.06 (d, *J* = 11.6 Hz). HRMS (ES+, *m*/*z*): calcd for (M + Na)^+^ C_18_H_33_F_2_N_2_O_6_PNa, 465.1950; found, 465.1942. HPLC (reverse-phase) 0.5 mL min^−1^ MeOH/H_2_O 70 : 30 in 12 min, *λ* = 210 nm, *R*_t_ = 5.82 min (100%).

### Di-*tert*-butyl 2,2′-((((*E*)-1,1-difluoro-5-hydroxy-4-methylpent-3-en-1-yl)phosphoryl)bis(azanediyl))(2*S*,2′*S*)-dipropionate (9c)

Yield: 81 mg (67%). ^1^H NMR (500 MHz, CDCl_3_): 5.54 (m, 1H, CH=C), 4.05 (s, 2H, CH_2_OH), 3.97 (m, 2H, 2 × CHNH), 3.53, 3.30 (m, 2 × 1H, 2 × NH), 2.89 (m, 2H, POCF_2_CH_2_), 2.30 (s, 1H, OH), 1.73 (s, 3H, CH_3_(CH_2_OH)C=CH), 1.47 (d, *J* = 4.0 Hz, 18H, 2 × OC(CH_3_)_3_), 1.40 (m, 6H, 2 × NHCHCH_3_). ^13^C NMR (125 MHz, CDCl_3_): 173.35, 142.15, 113.62, 82.38, 68.09, 49.47, 32.67, 27.92, 21.85, 14.04. ^31^P NMR (202 MHz, CDCl_3_): 13.19 (t, *J* = 96.3 Hz). ^19^F NMR (470 MHz, CDCl_3_): −108.85 (d, *J* = 7.7 Hz), −109.06 (d, *J* = 8.4 Hz). HRMS (ES+, *m*/*z*): calcd for (M + Na)^+^ C_20_H_37_F_2_N_2_O_6_PNa, 493.2260; found, 493.2255. HPLC (reverse-phase) 0.5 mL min^−1^ MeOH/H_2_O 70 : 30 in 12 min, *λ* = 210 nm, *R*_t_ = 5.81 min (100%).

### Dibenzyl 2,2′-((((*E*)-1,1-difluoro-5-hydroxy-4-methylpent-3-en-1-yl)phosphoryl)bis(azanediyl))(2*S*,2′*S*)-dipropionate (9d)

Yield: 76 mg (57%). ^1^H NMR (500 MHz, CDCl_3_): 7.34 (m, 10H, Ph), 5.51 (m, 1H, CH=C), 5.15 (m, 4H, 2 × OCH_2_C_6_H_5_), 4.13 (m, 2H, 2 × CHNH), 4.03 (s, 2H, CH_2_OH), 3.55, 3.35 (m, 2 × 1H, 2 × NH), 2.87 (m, 2H, POCF_2_CH_2_), 2.16 (s, 1H, OH), 1.70 (s, 3H, CH_3_(CH_2_OH)C=CH), 1.44, 1.37 (2 m, 2 ×3 H, 2 × NHCHCH_3_). ^13^C NMR (125 MHz, CDCl_3_): 173.76, 142.07, 135.15, 128.66, 128.54, 128.27, 113.38, 67.70, 64.38, 60.41, 48.83, 32.56, 31.34, 14.02. ^31^P NMR (202 MHz, CDCl_3_): 13.14 (t, *J* = 96.7 Hz). ^19^F NMR (470 MHz, CDCl_3_): −108.36 (d, *J* = 96.4 Hz), −108.94 (d, *J* = 59.3 Hz), −109.15 (d, *J* = 58.9 Hz), −109.72 (d, *J* = 96.7 Hz). HRMS (ES+, *m*/*z*): calcd for (M + Na)^+^ C_26_H_33_F_2_N_2_O_6_PNa, 561.1940; found, 561.1942. HPLC (reverse-phase) 0.5 mL min^−1^ MeOH/H_2_O 70 : 30 in 12 min, *λ* = 210 nm, *R*_t_ = 5.53 min (100%).

### Dimethyl 2,2′-((((*E*)-5-hydroxy-4-methylpent-3-en-1-yl)phosphoryl)bis(azanediyl))(2*S*,2′*S*)-dipropionate (14a)

Yield: 66 mg (63%). ^1^H NMR (500 MHz, CDCl_3_): 5.45 (m, 1H, CH=C), 4.05 (m, 2H, 2 × CHNH), 4.01 (s, 2H, CH_2_OH), 3.74, 3.73 (2 × s, 2 × 3H, 2 × OCH_3_), 3.02 (m, 2H, 2 × NH), 2.39 (m, 2H, PCH_2_CH_2_), 2.01 (s, 1H, OH), 1.79 (m, 2H, PCH_2_CH_2_), 1.65 (s, 3H, CH_3_(CH_2_OH)C=CH), 1.40 (m, 6H, 2 × NHCHCH_3_). ^13^C NMR (125 MHz, CDCl_3_): 175.33, 137.04, 124.07, 68.41, 52.44, 48.62, 29.64, 28.75, 21.62, 21.06, 13.80. ^31^P NMR (202 MHz, CDCl_3_): 29.05 (s). HRMS (ES+, *m*/*z*): calcd for (M + Na)^+^ C_14_H_27_N_2_O_6_PNa, 373.1498; found, 373.1504. HPLC (reverse-phase) 0.5 mL min^−1^ MeOH/H_2_O 70 : 30 in 12 min, *λ* = 210 nm, *R*_t_ = 5.80 min (98%).

### Diisopropyl 2,2′-((((*E*)-5-hydroxy-4-methylpent-3-en-1-yl)phosphoryl)bis(azanediyl))(2*S*,2′*S*)-dipropionate (14b)

Yield: 40 mg (33%). ^1^H NMR (500 MHz, CDCl_3_): 5.46 (m, 1H, CH=C), 5.01 (m, 2H, 2 × OCH(CH_3_)_2_), 4.00 (s, 2H, CH_2_OH), 3.97 (m, 2H, 2 × CHNH), 3.05 (m, 2H, 2 × NH), 2.38 (m, 2H, PCH_2_CH_2_), 1.80 (m, 2H, PCH_2_CH_2_), 1.70 (s, 3H, CH_3_(CH_2_OH)C=CH), 1.38 (m, 6H, 2 × NHCHCH_3_), 1.25 (m, 12H, 2 × OCH(CH_3_)_2_). ^13^C NMR (125 MHz, CDCl_3_): 174.39, 137.13, 124.14, 69.09, 68.44, 48.90, 29.78, 28.88, 21.89, 21.67, 21.12, 13.81. ^31^P NMR (202 MHz, CDCl_3_): 28.95 (s). HRMS (ES+, *m*/*z*): calcd for (M + Na)^+^ C_18_H_35_N_2_O_6_PNa, 429.2143; found, 429.2130. HPLC (reverse-phase) 0.5 mL min^−1^ MeOH/H_2_O 70 : 30 in 12 min, *λ* = 210 nm, *R*_t_ = 5.81 min (100%).

### Di-*tert*-butyl 2,2′-((((*E*)-5-hydroxy-4-methylpent-3-en-1-yl)phosphoryl)bis(azanediyl))(2*S*,2′*S*)-dipropionate (14c)

Yield: 43 mg (33%). ^1^H NMR (500 MHz, CDCl_3_): 5.45 (m, 1H, CH=C), 4.01 (s, 2H, CH_2_OH), 3.92 (m, 2H, 2 × CHNH), 3.02 (m, 2H, 2 × NH), 2.38 (m, 2H, PCH_2_CH_2_), 1.77 (m, 2H, PCH_2_CH_2_), 1.71 (s, 3H, CH_3_(CH_2_OH)C=CH), 1.46 (d, *J* = 4.6 Hz, 18H, 2 × OC(CH_3_)_3_), 1.36 (dd, *J* = 13.7, 7.1 Hz, 2 × NHCHCH_3_). ^13^C NMR (125 MHz, CDCl_3_): 174.12, 137.18, 124.27, 81.82, 68.52, 49.23, 29.82, 28.94, 27.98, 22.08, 21.16, 13.82. ^31^P NMR (202 MHz, CDCl_3_): 28.90 (s). HRMS (ES+, *m*/*z*): calcd for (M + Na)^+^ C_20_H_39_N_2_O_6_PNa, 457.2453; found, 457.2443. HPLC (reverse-phase) 0.5 mL min^−1^ MeOH/H_2_O 70 : 30 in 12 min, *λ* = 210 nm, *R*_t_ = 5.54 min (98%).

### Dibenzyl 2,2′-((((*E*)-5-hydroxy-4-methylpent-3-en-1-yl)phosphoryl)bis(azanediyl))(2*S*,2′*S*)-dipropionate (14d)

Yield: 128 mg (59%). ^1^H NMR (500 MHz, CDCl_3_): 7.34 (m, 10H, Ph), 5.40 (m, 1H, CH=C), 5.14 (m, 4H, 2 × OCH_2_C_6_H_5_), 4.06 (m, 2H, 2 × CHNH), 3.99 (s, 2H, CH_2_OH), 3.02 (m, 2H, 2 × NH), 2.34 (m, 2H, PCH_2_CH_2_), 1.74 (m, 2H, PCH_2_CH_2_), 1.67 (s, 3H, CH_3_(CH_2_OH)C=CH), 1.41, 1.32 (2d, 2 × 3H, *J* = 7.0 Hz, 2 × NHCHCH_3_). ^13^C NMR (125 MHz, CDCl_3_): 174.59, 136.99, 135.29, 128.64, 128.48, 128.25, 124.11, 68.41, 67.77, 48.76, 29.66, 28.75, 21.63, 21.05, 13.79. ^31^P NMR (202 MHz, CDCl_3_): 29.10 (s). HRMS (ES+, *m*/*z*): calcd for (M + Na)^+^ C_26_H_35_N_2_O_6_PNa, 525.2127; found, 525.2130. HPLC (reverse-phase) 0.5 mL min^−1^ MeOH/H_2_O 70:30 in 12 min, *λ* = 210 nm, *R*_t_ = 5.82 min (100%).

### Human serum stability assay

This experiment was carried out as previously reported.^20,26,40^ Briefly, 5.0 mg of the phosphonodiamidate ProPAgen 9b was dissolved in a mixture of 0.05 mL of DMSO and 0.15 mL D_2_O. After recording the first ^31^P NMR data, 0.3 mL human serum (Merck Life Sciences) was added and monitored by NMR. The experiment was run on NMR ^31^P mode and scanned every half an hour for a total of 7.5 h. The incubation temperature was 37 °C. Recorded data were processed and analyzed with Bruker Topspin 2.1 software.

### Carboxypeptidase Y assay

This experiment was carried out as previously reported.^20,26,40^ 5.0 mg of the phosphonodiamidate ProPAgen 9b was dissolved in 0.2 mL of acetone, and 0.4 mL of Trizma buffer (pH 7.4) was added followed by 0.5 mg carboxypeptidase Y in 0.2 ml Trizma buffer (pH 7.4). The experiment was run on NMR phosphorus mode and scanned every half an hour for 6.5 h. The incubation temperature was 37 °C. Recorded data were processed and analyzed with Bruker Topspin 2.1 software.

### *In vitro* Vγ9/Vδ2 T cell activation assay

Healthy donor peripheral blood mononuclear cells (PBMCs) were harvested as previously described.^20^ To assess the activation of Vγ9/Vδ2 T cells, PBMCs were seeded into U-bottom tissue culture-treated 96-well plates at a cell density of 500 000 cells per well. The cells were incubated with medium-only (control), in the presence of zoledronate at 10 pM to 100 μM, and with HMBP ProPAgens initially at 10 pM to 100 μM, and then at 1 aM to 100 μM for compounds 9d and 14d in a separate experiment. The cells were incubated at 37 °C/5% CO_2_ overnight, and stained by flow cytometry for the following markers: viability (Zombie Aqua 1 : 400), CD3 (BV421 1 : 100), CD8 (BV650 1 : 200), Vγ9 (PEcy5 1 : 400), Vδ2 (APC 1 : 200), CD69 (PE 1 : 25), CD25 (FITC 1 : 100). The samples were acquired *via* LSRFortessa X20 (BD Biosciences), and the data obtained were analyzed using FlowJo v10 and GraphPad Prism v9 software.

### Cytotoxicity assay

To assess the level of killing of drug-treated and untreated tumour cells, a Europium-based cytotoxicity assay was performed (DELFIA, Perkin Elmer) as described elsewhere.^47^ Target tumour T24 cells (human bladder carcinoma) were cultured in PBS for 2 h at 37 °C/5% CO_2_ in the following conditions: untreated, 10 μM zoledronate, 10 nM 9d and 14d. The cells were then washed 3 times at 600 × *g* for 5 min in PBS to remove any excess drug and incubated in PBS with BATDA labelling agent (used at 1 μl per mL) for 20 min at 37 °C as before. In the meantime, Vγ9/Vδ2 T cells, previously expanded with 5 μM zoledronate and 100 U mL^−1^ IL2 over a period of 14 days, were thawed, counted and re-suspended to a cell concentration of 4 × 106 cells per mL. After the BATDA labelling, T24 cells were washed 3 times in medium at 4 °C and re-suspended to a concentration of 5 × 10^4^ cells per mL. 100 μl of T24 cells were then seeded into U-bottom tissue culture 96-well plates and co-cultured with 100 μL per well Vγ9/Vδ2 T cells (*i.e.* effector : target ratio 80 : 1) as previously described.^47^ Drug-treated and untreated T24 cells were also seeded alone without Vγ9/Vδ2 effectors and 100 μL media was added per well instead. To the positive killing control, 10% v/v lysis buffer was added to drug-untreated T24 cells. The plate was centrifuged at 200 × *g* for 2 min to bring cells into contact in the co-cultures, and the plate with all samples was incubated at 37 °C/5% CO_2_ for 1 h. Following this incubation, the plate was centrifuged again at 600 × *g* for 2 min and 25 μL of the supernatant was transferred into a flat-bottom 96-well optical plate, to which Europium solution was added at 200 μL per well. Time-resolved fluorescence was then measured using a PHERAstar microplate reader (BMG Labtech). Specific lysis (% killing of T24 cells) was calculated as follows: [(experimental release − spontaneous release)/(maximal release − spontaneous release)] × 100. The data were processed and analysed using Microsoft Excel and GraphPad Prism v9 software.

## Abbreviations

Bn: Benzyl
BTN2A1: Butyrophilin 2A1
BTN3A1: Butyrophilin 3A1
clog *P*: Calculated log *P*
(COCl)_2_: Oxalyl chloride
DCM: Dichloromethane
Et_3_N: Triethylamine
(EtO)_3_P: Triethyl phosphite
HMBP: (*E*)-4-Hydroxy-3-methylbut-2-enyl monophosphate
HMBPP: (*E*)-4-Hydroxy-3-methylbut-2-enyl pyrophosphate
HPMA: Hexamethylphosphoramide
IPP: Isopentenyl pyrophosphate
*i*Pr: Isopropyl
LDA: Lithium diisopropylamine
Me: Methyl
PAg: Phosphoantigen
ProPAgen: Prodrug of a phosphoantigen
*t*Bu: *tert*-Butyl
TCR: T cell receptor
TMSBr: Trimethylsilyl bromide

## Author contributions

Q. X. synthesized the compounds reported in this work. E. J. carried out the *in vitro* serum stability and metabolism studies. M. S. conducted the biological evaluation of the compounds. J. D. the confirmatory mass spectrometry and purity of the final Prodrugs. Y. M., J. H. R. T., and B. E. W. designed the experiments and supervised the work. The manuscript was written through contributions of all authors, and all of the authors have given approval to the final version of the manuscript.

## Conflicts of interest

Y. M. and B. E. W. are named investors in a patent application covering the prodrugs presented in this work (application number: EP23171966.7). Also, Y. M. and B. E. W. provide consultancy services regarding the development of gamma delta T cell based immunotherapy approaches.

## Supplementary Material

MD-015-D4MD00208C-s001
