# Supplementary material for: Phosphonodiamidate prodrugs of phosphoantigens (ProPAgens) exhibit potent Vγ9/Vδ2 T cell activation and eradication of cancer cells
Source: RSC Med Chem. 2024 Jun 3;15(7):2462–73. doi: 10.1039/d4md00208c (PMC11253855; doi:10.1039/d4md00208c)
Supplement: MD-015-D4MD00208C-s001 [file MD-015-D4MD00208C-s001.pdf]

Electronic Supplementary Material (ESI) for RSC Medicinal Chemistry.
This journal is © The Royal Society of Chemistry 2024

Phosphonodiamidate Prodrugs of Phosphoantigens (ProPAgens) Exhibit Potent Vγ9/Vδ2 T Cell Activation and Eradication of Cancer Cells

Qin Xu,[1,‡] Maria Sharif,[2,3,‡] Edward James,[1] Jack O. Dismorr,[4] James H. R. Tucker,[4] Benjamin E. Willcox[2,3,*], and Youcef Mehellou[1,5*]

[1]*School of Pharmacy and Pharmaceutical Sciences, Cardiff University, Cardiff CF10 3NB, U.K.*
[2]*Institute of Immunology and Immunotherapy, University of Birmingham, Birmingham B15 2TT, U.K.*
[3]*Cancer Immunology and Immunotherapy Centre, University of Birmingham, Birmingham B15 2TT, U.K.*
[4]*School of Chemistry, University of Birmingham, Birmingham B15 2TT, U.K.*
[5]Medicines Discovery Institute, *Cardiff University, Cardiff CF10 3AT, U.K.*

**Corresponding Authors: B.E.W: b.willcox@bham.ac.uk, YM; MehellouY1@cardiff.ac.uk,*

Content

- Figure S1: HPLC chromatograms of final ProPAgens……………………………………………………………….S2-S9
- Figure S2: Activation of Vγ9/Vδ2 T cells by compounds 9d and 14d ……………………………………….S10
- Figure S3: HPLC chromatograms of ProPAgens 9d and 14d after the assays……………………..…….S11-S14
- Figure S4: Background activation of Vγ9/Vδ2 T cells with DMSO and activation of CD8+ T cells with compounds 9d and 14d……………………………………………………………………………………………………S15

Figure S1. HPLC chromatograms of final ProPAgens.

Analysis Report

<Sample Information>

ProPAgen 9a

Sample Name	: DAFN-Me-1		
Sample ID	: Me01		
Data Filename	: XQ-DAFN-Me-1.lcd		
Method Filename	: method diamidate.lcm		
Batch Filename	: Batch Meisotbu01 310119.lcb		
Vial #	: 1-1	Sample Type	: Unknown
Injection Volume	: 10 uL		
Date Acquired	: 31/01/2019 14:55:21	Acquired by	: System Administrator
Date Processed	: 31/01/2019 15:07:21	Processed by	: System Administrator

<Chromatogram>

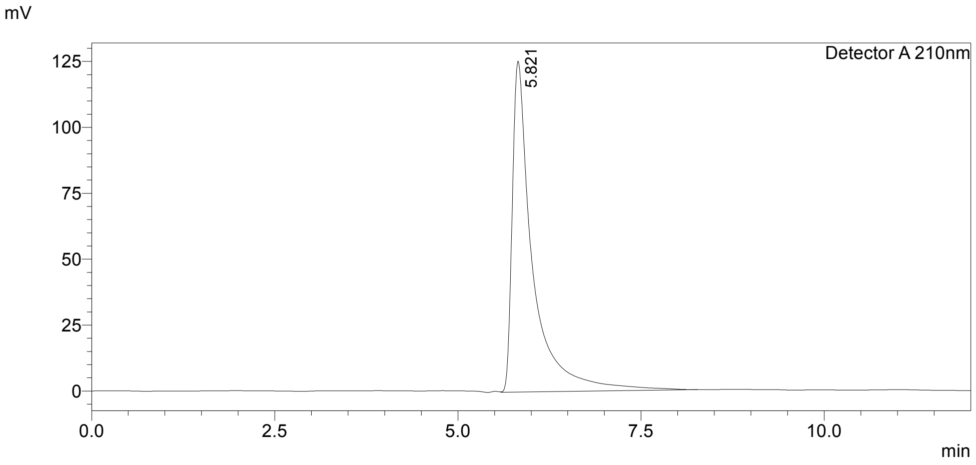

<Peak Table>

Detector A 210nm

Peak#	Ret. Time	Area	Height	Area%
1	5.821	2444504	125392	100.000
Total		2444504	125392	100.000

Analysis Report

<Sample Information>

ProPAgen 9b

Sample Name : DAFN-Iso-1
Sample ID : Iso01
Data Filename : XQ-DAFN-Iso-1.lcd
Method Filename : method diamidate.lcm
Batch Filename : Batch Meisotbu01 310119.lcb
Vial # : 1-2
Injection Volume : 10 uL
Date Acquired : 31/01/2019 15:07:40
Date Processed : 31/01/2019 15:19:41

Sample Type : Unknown

Acquired by : System Administrator
Processed by : System Administrator

<Chromatogram>

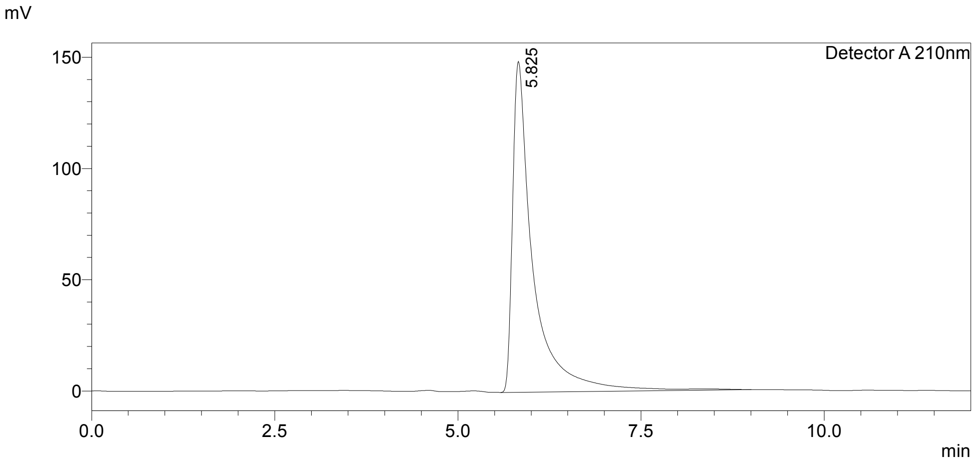

<Peak Table>

Detector A 210nm

Peak#	Ret. Time	Area	Height	Area%
1	5.825	2891023	148697	100.000
Total		2891023	148697	100.000

Analysis Report

<Sample Information>

ProPAgen 9c

Sample Name	: tBu-004		
Sample ID	: tBu004		
Data Filename	: XQ-DAFN-tBU-004.lcd		
Method Filename	: method diamidate.lcm		
Batch Filename	: batch05 diamidate 040219.lcb		
Vial #	: 1-1	Sample Type	: Unknown
Injection Volume	: 5 uL		
Date Acquired	: 04/02/2019 15:35:14	Acquired by	: System Administrator
Date Processed	: 04/02/2019 15:47:14	Processed by	: System Administrator

<Chromatogram>

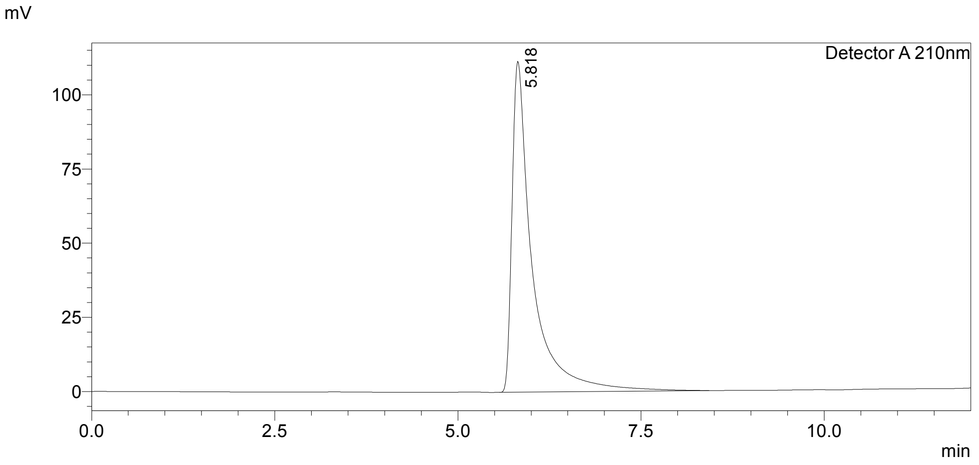

<Peak Table>

Detector A 210nm

Peak#	Ret. Time	Area	Height	Area%
1	5.818	2145865	111485	100.000
Total		2145865	111485	100.000

Analysis Report

<Sample Information>

ProPAgen 9d

Sample Name	: DAFN-BN-001
Sample ID	: BN-001
Data Filename	: XQ-DAFN-Bn-2.lcd
Method Filename	: method diamidate.lcm
Batch Filename	: Batch 0.7mlmin 310119.lcb
Vial #	: 1-1
Injection Volume	: 10 uL
Date Acquired	: 31/01/2019 14:16:14
Date Processed	: 31/01/2019 14:28:15
Sample Type	: Unknown
Acquired by	: System Administrator
Processed by	: System Administrator

<Chromatogram>

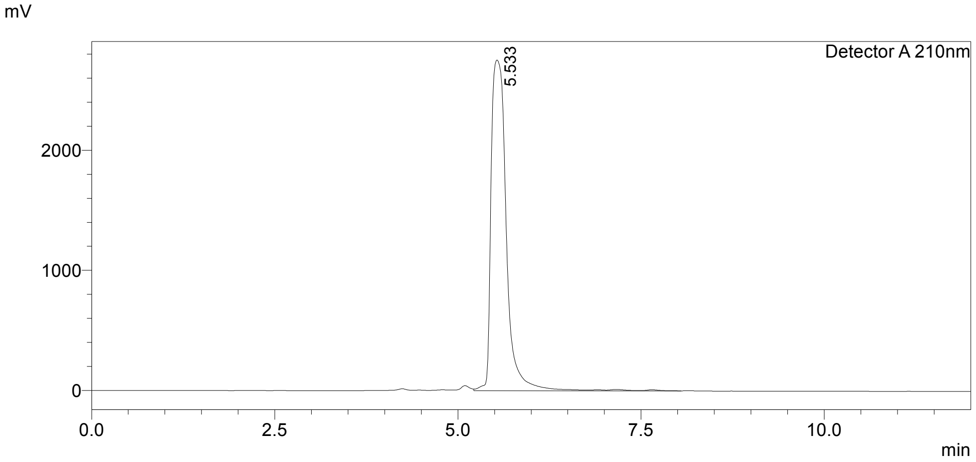

<Peak Table>

Detector A 210nm

Peak#	Ret. Time	Area	Height	Area%
1	5.533	40083588	2753823	100.000
Total		40083588	2753823	100.000

Analysis Report

<Sample Information>

ProPAgen 14a

Sample Name	: NDFN-Me-001		
Sample ID	: NDFN-Me-001		
Data Filename	: XQ-NDFN-Me-002.lcd		
Method Filename	: method diamidate.lcm		
Batch Filename	: Batch02 diamidate 110219.lcb		
Vial #	: 1-1	Sample Type	: Unknown
Injection Volume	: 5 uL		
Date Acquired	: 11/02/2019 14:48:12	Acquired by	: System Administrator
Date Processed	: 11/02/2019 15:00:14	Processed by	: System Administrator

<Chromatogram>

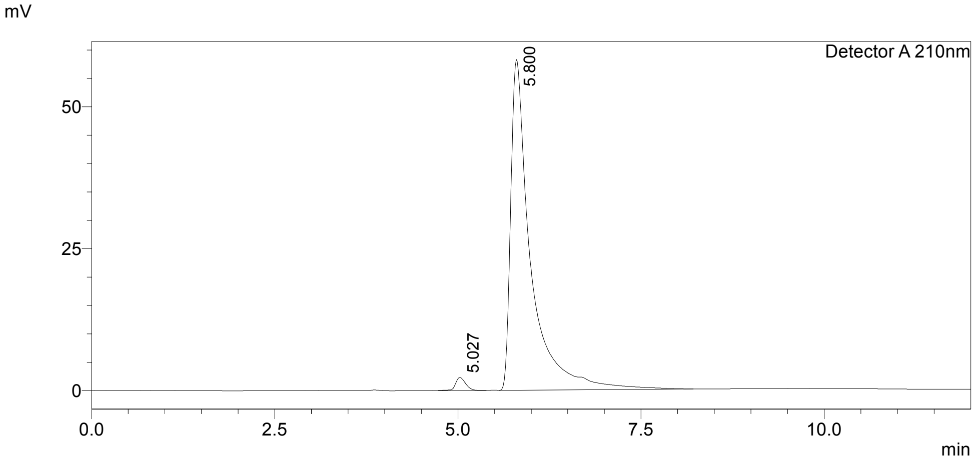

<Peak Table>

Detector A 210nm

Peak#	Ret. Time	Area	Height	Area%
1	5.027	22379	2294	1.976
2	5.800	1110111	58194	98.024
Total		1132490	60488	100.000

Analysis Report

<Sample Information>

ProPAgen 14b

Sample Name	: NDFN-Iso001
Sample ID	: Iso001
Data Filename	: XQ-NDFN-ISO-01.lcd
Method Filename	: method diamidate.lcm
Batch Filename	: Batch01 diamidate 040219.lcb
Vial #	: 1-2
Injection Volume	: 10 uL
Date Acquired	: 04/02/2019 14:37:18
Date Processed	: 04/02/2019 14:49:19
Sample Type	: Unknown
Acquired by	: System Administrator
Processed by	: System Administrator

<Chromatogram>

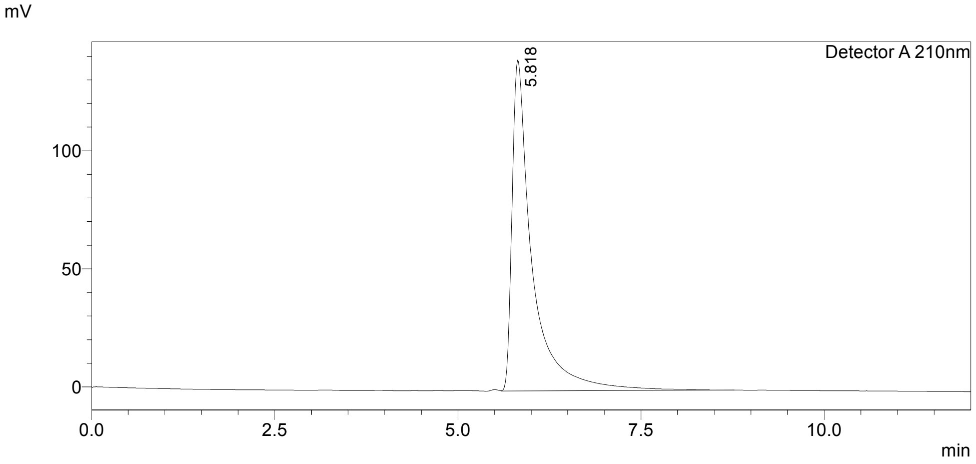

<Peak Table>

Detector A 210nm

Peak#	Ret. Time	Area	Height	Area%
1	5.818	2693580	140021	100.000
Total		2693580	140021	100.000

Analysis Report

<Sample Information>

ProPAgen 14c

Sample Name	: NDFN-tBu-001		
Sample ID	: NDFN-tBu-001		
Data Filename	: XQ-NDFN-tBu-003.lcd		
Method Filename	: method diamidate.lcm		
Batch Filename	: batch4 diamidate 110219.lcb		
Vial #	: 1-1	Sample Type	: Unknown
Injection Volume	: 10 uL		
Date Acquired	: 11/02/2019 15:23:12	Acquired by	: System Administrator
Date Processed	: 11/02/2019 15:40:57	Processed by	: System Administrator

<Chromatogram>

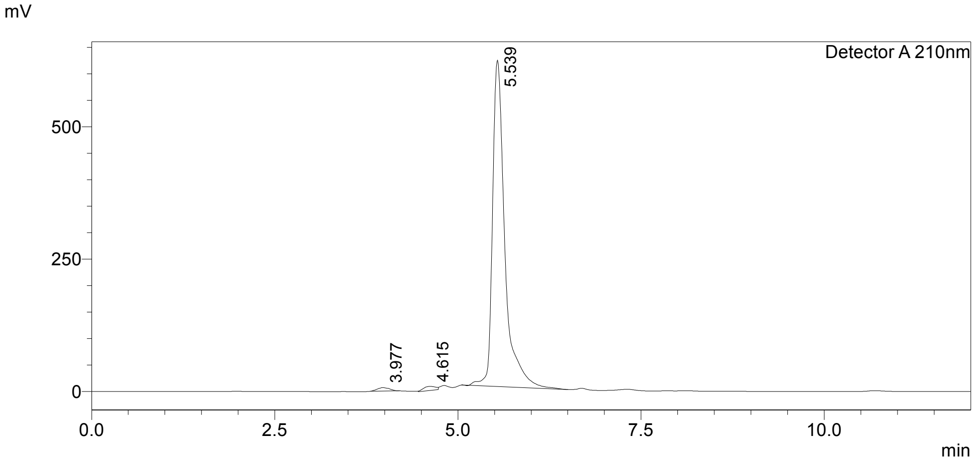

<Peak Table>

Detector A 210nm

Peak#	Ret. Time	Area	Height	Area%
1	3.977	76494	6656	1.035
2	4.615	92632	7718	1.253
3	5.539	7223345	616035	97.712
Total		7392471	630409	100.000

C:\LabSolutions\Data\YM\XQ\XQ-NDFN-tBu-003.lcd

Analysis Report

<Sample Information>

ProPAgen 14d

Sample Name	: NDFN-Bn001		
Sample ID	: Bn-01		
Data Filename	: XQ-NDFN-Bn001.lcd		
Method Filename	: method diamidate.lcm		
Batch Filename	: Batch01 diamidate 050219.lcb		
Vial #	: 1-3	Sample Type	: Unknown
Injection Volume	: 10 uL		
Date Acquired	: 05/02/2019 12:50:17	Acquired by	: System Administrator
Date Processed	: 05/02/2019 13:02:18	Processed by	: System Administrator

<Chromatogram>

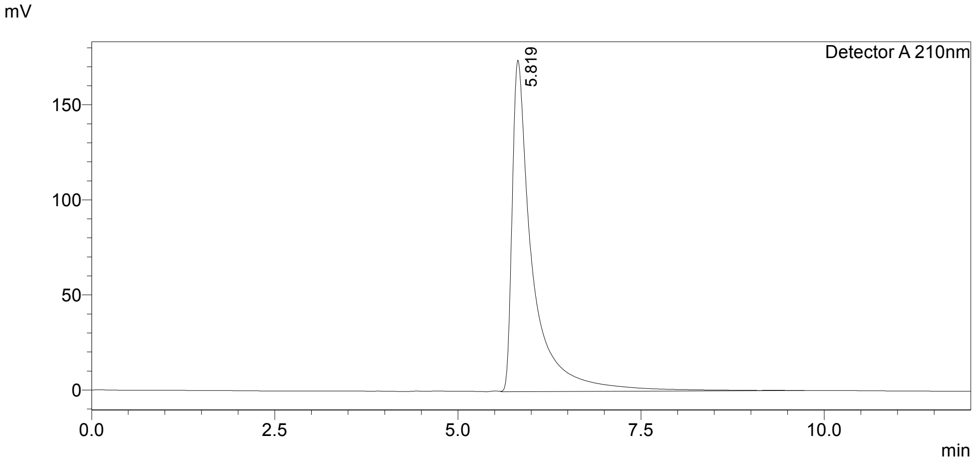

<Peak Table>

Detector A 210nm

Peak#	Ret. Time	Area	Height	Area%
1	5.819	3388393	174211	100.000
Total		3388393	174211	100.000

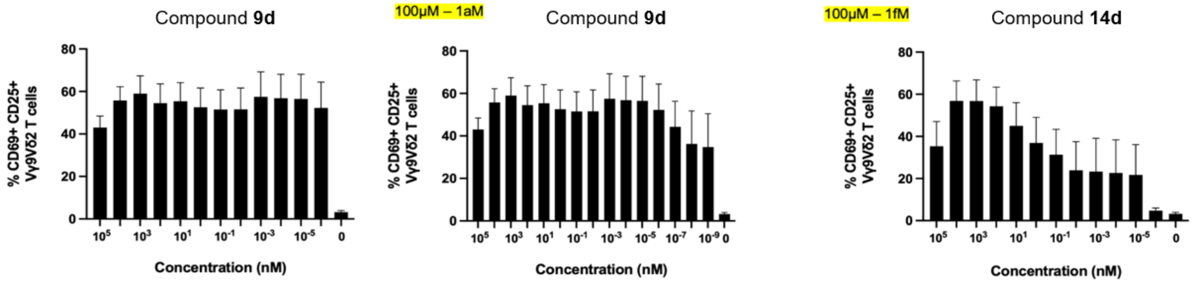

Figure S2. Phosphonodiamidate ProPAgen 9d- and 14d-mediated activation of Vγ9/Vδ2 T cells after overnight incubation of PBMC with 9d at 1 attomolar (aM) to 100µM and 14d at 1 femtomolar (fM) to 100µM in order to determine the EC_{50} values of these compounds. The level of activation is presented as % CD69+ CD25+ Vγ9/Vδ2 T cells. Data was analyzed with GraphPad Prism v9 software and is shown as mean ± SE (n=4).

Figure S3. HPLC traces of **ProPAgens 9d** and **14d** following dissolution and storage.

```
Data File C:\Chem32\3\Data\Jack\Jack-2022_09_07_3 2022-09-07 17-20-43\DHFN BN stock 5uL.D
Sample Name: DHFN BN stock 5uL

     =====================================================================
     Acq. Operator   : SYSTEM                         Seq. Line :    4
     Acq. Instrument : LC-FC                           Location :    2
     Injection Date  : 07-Sep-22 6:39:51 PM                 Inj :    1
                                                     Inj Volume : 15.000 µl
     Different Inj Volume from Sample Entry!  Actual Inj Volume : 5.000 µl
     Method          : C:\Chem32\3\Data\Jack\Jack-2022_09_07_3 2022-09-07 17-20-43\7030 MeOHH2O
                       12min.M (Sequence Method)
     Last changed    : 05-Sep-22 10:33:20 AM by SYSTEM
     Sample Info     : DHFN BN stock 5uL

     Additional Info : Peak(s) manually integrated
```

ProPAgen 9d

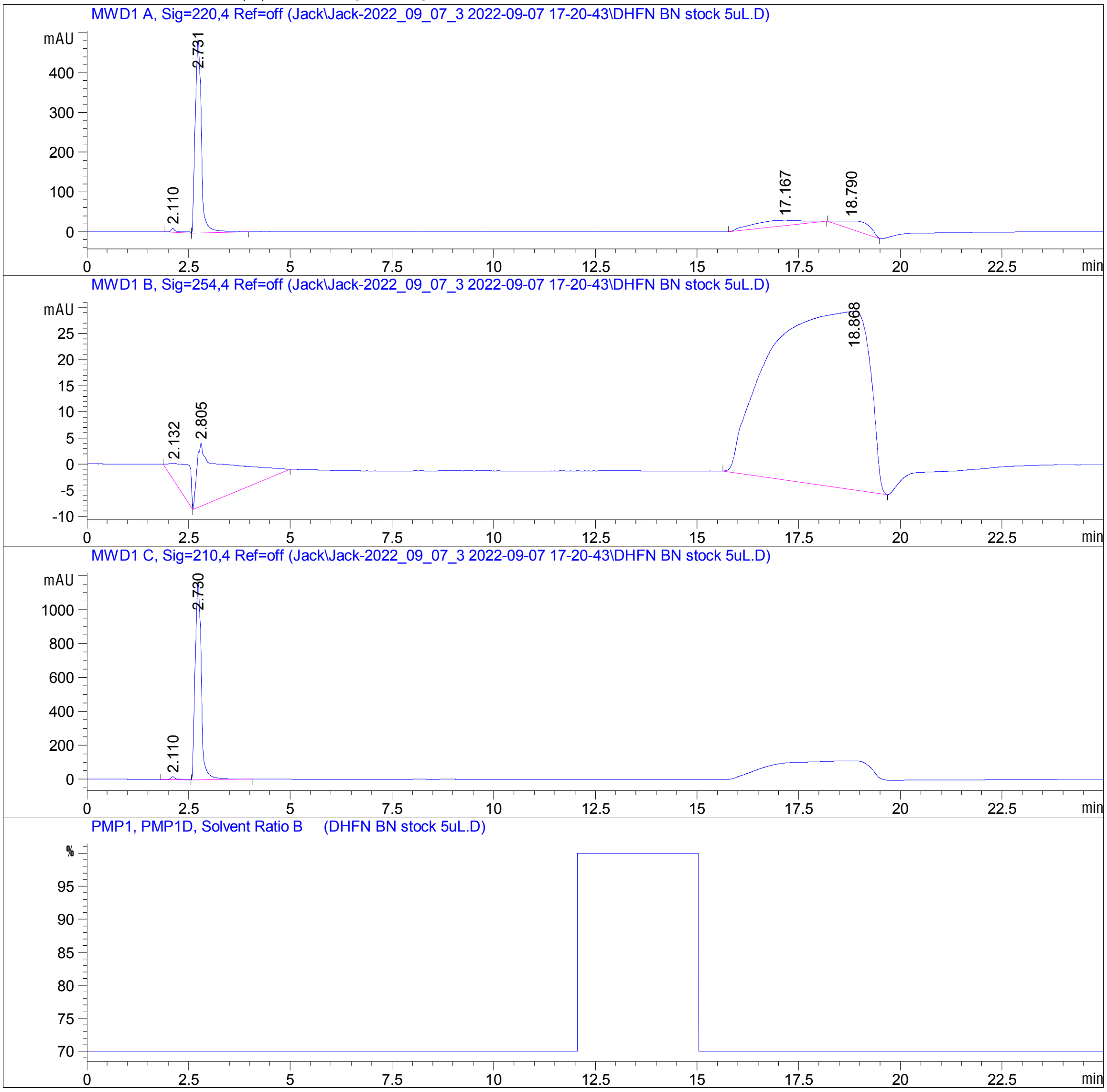


```
     ==================================================================================
                                Fraction Information
```

===
No Fractions found.
===

ProPAgen 9d

Area Percent Report

Sorted By : Signal
Multiplier : 1.0000
Dilution : 1.0000
Do not use Multiplier & Dilution Factor with ISTDs

Signal 1: MWD1 A, Sig=220,4 Ref=off

Peak #	RetTime [min]	Type	Width [min]	Area [mAU*s]	Height [mAU]	Area %
1	2.110	BB	0.1661	121.78243	9.72608	1.5731
2	2.731	BB	0.1415	5120.33057	483.71109	66.1404
3	17.167	BB	1.1526	1311.39526	13.34365	16.9396
4	18.790	BB	0.6911	1188.10461	20.29043	15.3470
Totals :				7741.61288	527.07125	

Signal 2: MWD1 B, Sig=254,4 Ref=off

Peak #	RetTime [min]	Type	Width [min]	Area [mAU*s]	Height [mAU]	Area %
1	2.132	BB	0.6362	172.38980	3.27453	2.6891
2	2.805	BB	0.6326	642.50555	12.07816	10.0225
3	18.868	BB	1.9244	5595.75146	34.13763	87.2884
Totals :				6410.64682	49.49033	

Signal 3: MWD1 C, Sig=210,4 Ref=off

Peak #	RetTime [min]	Type	Width [min]	Area [mAU*s]	Height [mAU]	Area %
1	2.110	BB	0.1220	154.47354	17.67632	1.2531
2	2.730	BB	0.1421	1.21732e4	1162.92981	98.7469
Totals :				1.23277e4	1180.60613	

===
*** End of Report ***

Data File C:\Chem32\3\Data\Jack\Jack-2022_09_05 2022-09-05 10-44-34\NDFN BN 1.D
Sample Name: NDFN BN 1

ProPAgen 14d

===

Acq. Operator : SYSTEM　　Seq. Line : 8
Acq. Instrument : LC-FC　　Location : 13
Injection Date : 05-Sep-22 1:47:27 PM　　Inj : 1
Inj Volume : 15.000 µl
Different Inj Volume from Sample Entry! Actual Inj Volume : 5.000 µl
Method : C:\Chem32\3\Data\Jack\Jack-2022_09_05 2022-09-05 10-44-34\7030 MeOHH2O 12min.M (Sequence Method)
Last changed : 05-Sep-22 10:33:20 AM by SYSTEM
Sample Info : NDFN BN 1

Additional Info : Peak(s) manually integrated

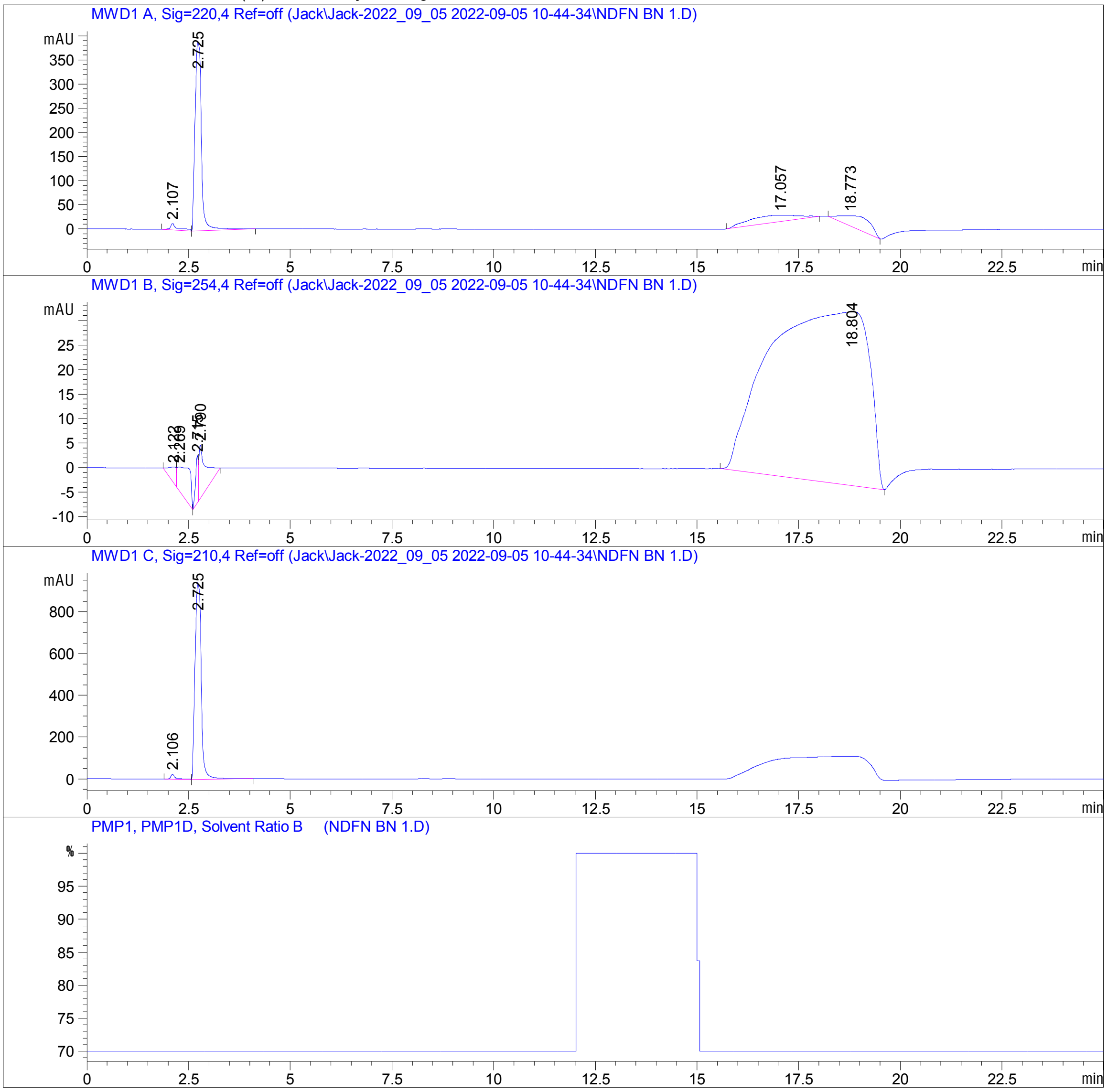

===

Fraction Information

===
No Fractions found.
===
===
===

ProPAgen 14d

Area Percent Report

===

Sorted By : Signal
Multiplier : 1.0000
Dilution : 1.0000
Do not use Multiplier & Dilution Factor with ISTDs

Signal 1: MWD1 A, Sig=220,4 Ref=off

Peak #	RetTime [min]	Type	Width [min]	Area [mAU*s]	Height [mAU]	Area %
1	2.107	BB	0.1696	167.34492	13.04740	2.4087
2	2.725	BV R	0.1439	4313.51758	393.42148	62.0863
3	17.057	BV R	1.1181	1212.06775	12.80741	17.4458
4	18.773	BB	0.7254	1254.68115	20.51801	18.0592

Totals : 6947.61140 439.79429

Signal 2: MWD1 B, Sig=254,4 Ref=off

Peak #	RetTime [min]	Type	Width [min]	Area [mAU*s]	Height [mAU]	Area %
1	2.122	BV	0.1744	40.23585	3.08034	0.6442
2	2.269	VB	0.3392	130.60985	4.77356	2.0911
3	2.715	BV	0.0656	42.26519	9.54859	0.6767
4	2.790	VB	0.1572	132.64157	10.80398	2.1236
5	18.804	BB	1.9574	5900.34521	35.38522	94.4645

Totals : 6246.09768 63.59169

Signal 3: MWD1 C, Sig=210,4 Ref=off

Peak #	RetTime [min]	Type	Width [min]	Area [mAU*s]	Height [mAU]	Area %
1	2.106	BB	0.1224	203.74788	23.20883	1.9814
2	2.725	BB	0.1406	1.00791e4	943.64679	98.0186

Totals : 1.02828e4 966.85562

===
*** End of Report ***

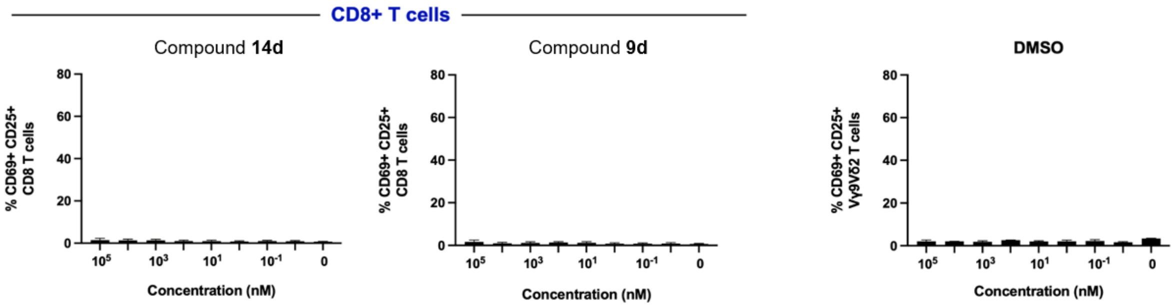

Figure S4. Phosphonodiamidate ProPAgen 9d- and 14d-mediated activation of CD8+ T cells (left) in the same overnight cultures as Vγ9/Vδ2 T cells (mean ± SE; n=4). Level of Vγ9/Vδ2 T cell activation with DMSO (right) diluted in the same way as the ProPAgens (mean ± SE; n=4). Data was analyzed with GraphPad Prism v9 software.
